# TiO_2_ Electron Transport Layer with p–n Homojunctions for Efficient and Stable Perovskite Solar Cells

**DOI:** 10.1007/s40820-024-01407-3

**Published:** 2024-05-03

**Authors:** Wenhao Zhao, Pengfei Guo, Jiahao Wu, Deyou Lin, Ning Jia, Zhiyu Fang, Chong Liu, Qian Ye, Jijun Zou, Yuanyuan Zhou, Hongqiang Wang

**Affiliations:** 1 State Key Laboratory of Solidification Processing, Center for Nano Energy Materials, School of Materials Science and Engineering, Northwestern Polytechnical University and Shaanxi Joint Laboratory of Graphene (NPU), Xi’an, 710072 People’s Republic of China; 2 Department of Chemical and Biological Engineering, Hong Kong University of Science and Technology, Clear Water Bay, Hong Kong SAR, People’s Republic of China; 3 Key Laboratory for Green Chemical Technology of the Ministry of Education, School of Chemical Engineering and Technology, Tianjin University, Tianjin, 300072 People’s Republic of China; 4 Chongqing Innovation Center of Northwestern, Polytechnical University, Northwestern Polytechnical University, Chongqing, 401135 People’s Republic of China

**Keywords:** Electron transport layer, p–n homojunction, Electron mobility, Buried interface, Perovskite solar cells

## Abstract

Low-temperature processed electron transport layer (ETL) of TiO_2_ that is widely used in planar perovskite solar cells (PSCs) has inherent low carrier mobility, resulting in insufficient photogenerated electron transport and thus recombination loss at buried interface. Herein, we demonstrate an effective strategy of laser embedding of p-n homojunctions in the TiO_2_ ETL to accelerate electron transport in PSCs, through localized build-in electric fields that enables boosted electron mobility by two orders of magnitude. Such embedding is found significantly helpful for not only the enhanced crystallization quality of TiO_2_ ETL, but the fabrication of perovskite films with larger-grain and the less-trap-states. The embedded p–n homojunction enables also the modulation of interfacial energy level between perovskite layers and ETLs, favoring for the reduced voltage deficit of PSCs. Benefiting from these merits, the formamidinium lead iodide (FAPbI_3_) PSCs employing such ETLs deliver a champion efficiency of 25.50%, along with much-improved device stability under harsh conditions, i.e., maintain over 95% of their initial efficiency after operation at maximum power point under continuous heat and illumination for 500 h, as well as mixed-cation PSCs with a champion efficiency of 22.02% and over 3000 h of ambient storage under humidity stability of 40%. Present study offers new possibilities of regulating charge transport layers via p-n homojunction embedding for high performance optoelectronics.

**Supplementary Information:**

The online version contains supplementary material available at 10.1007/s40820-024-01407-3.

## Highlights

- Developing a universal strategy of the p–n homojunction engineering that could significantly boost electron mobility of electron transport layer (ETL) by two orders of magnitude.
- Proposing a new mechanism based on p–n homojunction to explain inhibited carrier loss at buried interface.
- Setting a new performance benchmark as high as 25.50% for planar perovskite solar cells employing TiO_2_ as ETLs.

**Supplementary Information:**

The online version contains supplementary material available at 10.1007/s40820-024-01407-3.

## Graphical abstract

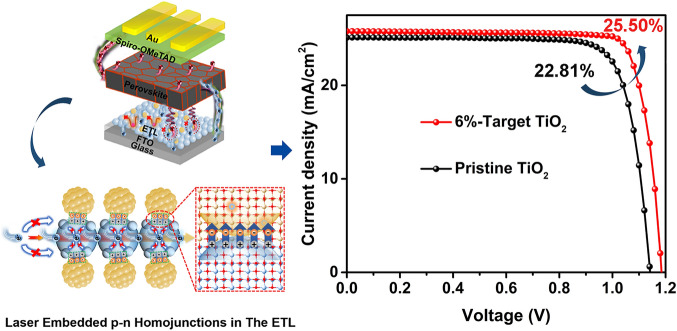

## Introduction

Planar metal halide perovskite solar cells (PSCs) have been pushing the record-breaking power conversion efficiencies (PCEs) over 26%, as well as durable stability and compatibility with large-scale manufacture [1–3]. The reasons behind such significant achievements are associated with the strategies of integrating low-bandgap and less-trap-state formamidine-based perovskite with widely regarded as efficient charge transport layers [4]. In particular, regular planar PSCs indispensably necessitate high-quality and compatible electron transport layer (ETL) owing to their high light transmittance, suitable energy level, and low-temperature processability, which are crucial to not only the growth of the top perovskite grain but also extraction and collection of the photogenerated electrons to the electrode [5]. While noting that widely adopted metal oxide ETLs are inevitably endowed with inherently inferior electron mobility (for example, TiO_2_: usually at the level of 10^–5^ ~ 10^–4^ cm^2^ V^−1^ s^−1^) due to low-temperature process that could arouse charge carrier accumulation and recombination loss at buried interface, resulting in less-than-ideal efficiency and unrobust environmental stability [6]. Several state-of-the-art engineering strategies have been developed to address the imperfections of such ETLs, as exemplified by employing nitrogen-doped TiO_2_ ETLs with reduced the electrical series resistance, as well as high-lattice-matching SrSnO_3_ as the ETLs enabling ordered beginning of the growth of perovskite to synchronously rule the buried defects and carrier dynamics in PSCs [7, 8].

Creation of p–n junction either in the active layer or at upper/buried interface has been exploited as an effective strategy to tune the charge carrier transport in PSCs [9, 10]. For example, embedding 2D graphdiyne or 0D fluorinated-gold-cluster at grain boundaries within the perovskite films enables the construction of p-n heterojunctions, which provides an extra channel to favor the exciton separation and charge transport [11, 12]. It is reported that the TiO_2_ with Ti vacancies shows inherent p-type conductivity with high charge mobility, demonstrating a nearly sevenfold increase over the normal n-type TiO_2_ [13]. Motivated by embedding p–n junction in perovskite to alleviate the carrier loss, it would thus be highly promising to construct the p–n homojunction in the ETLs by introducing Ti-defected TiO_2_ in n-type TiO_2_ for further pronounced electron conducting capability and highly efficient and stable TiO_2_-based planar PSCs. The challenge however remains on not only the technical embedding of such p-type TiO_2_ in n-type TiO_2_ ETLs, but also the understanding of such homojunction influencing the carriers transport in the ETL.

**Scheme 1:**
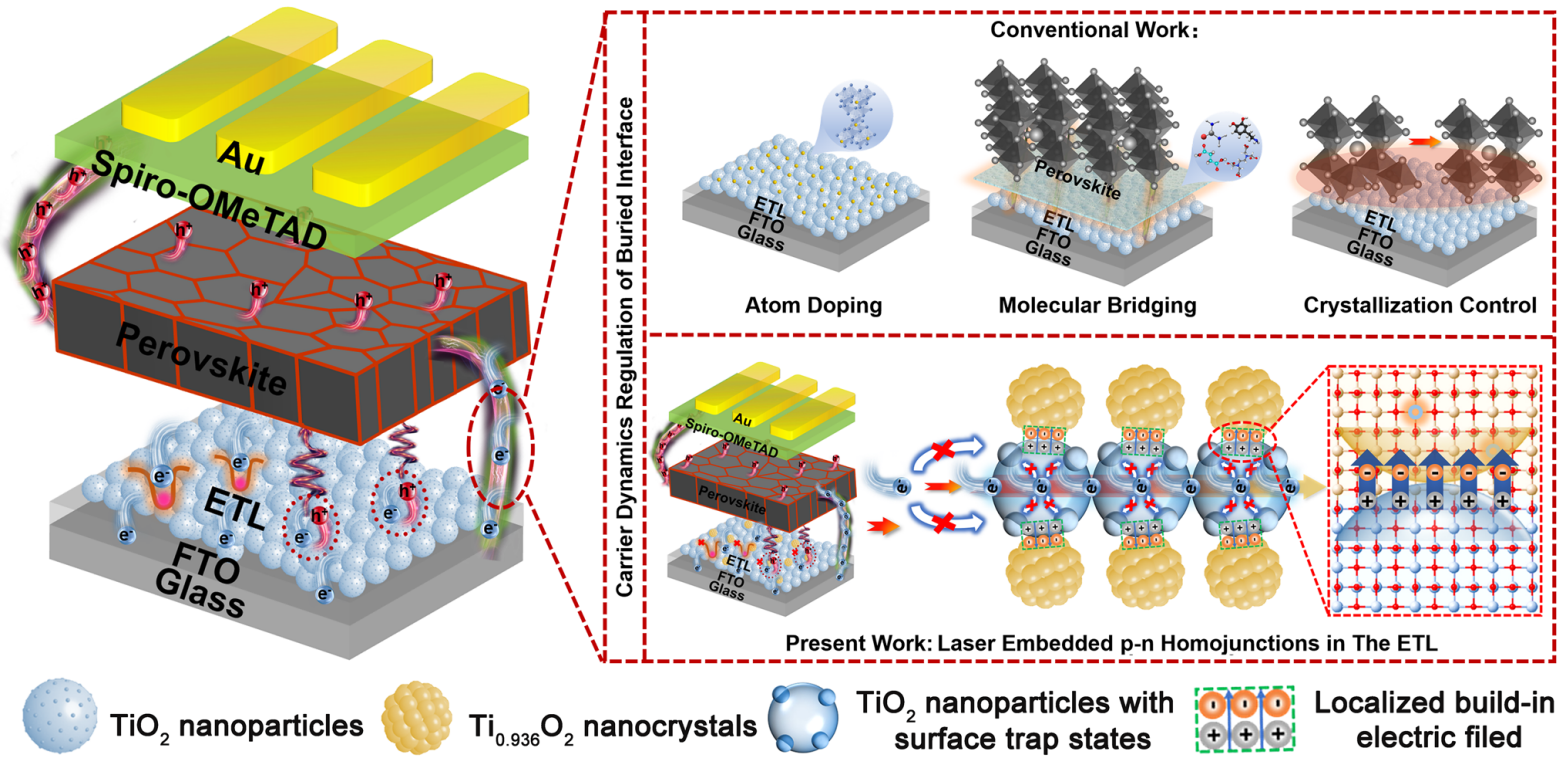
Schematic illustration of the effects of embedding coherent p–n homojunctions on carrier dynamics at buried interface compared with those of conventional works [7, 8, 16]

Present work demonstrates an effective strategy of constructing Ti_0.936_O_2_@TiO_2_-based p–n homojunction to improve electron mobility and photovoltaic performance of planar PSCs through embedding laser-derived p-type Ti_0.936_O_2_ in TiO_2_ ETL. Such embedding of Ti-defected TiO_2_ could modulate the crystallization kinetics of the TiO_2_ matrix by restraining the rutile phase that is detrimental to light stability of PSCs, contributing to the formation of high-quality TiO_2_ ETLs. The formed p-n homojunction enables also not only elimination of the interfacial lattice distortion between Ti_0.936_O_2_ and TiO_2_, but also more efficient transport of charge carriers at both surfaces and boundaries of TiO_2_ ETLs through localized build-in electric fields, thus reducing the recombination loss (Scheme 1). More importantly, such a novel Ti_0.936_O_2_@TiO_2_ composite ETL has also exerted a significant influence on the construction of less-trap-states and larger-grain perovskite films. Benefiting from these merits, we obtain highly efficient formamidinium lead iodide (FAPbI_3_) PSCs with PCE up to 25.50%, which ranks as far as we know among the top in records of TiO_2_-based planar PSCs. Owing to the synchronous regulation of the Ti_0.936_O_2_ in photocatalytic activity of TiO_2_ ETLs and film quality of perovskite layers, we have also obtained highly stable FAPbI_3_ PSC that maintains over 95% of their initial efficiency at maximum power point under continuous illumination for 500 h, as well as mixed-cation PSCs with pronounced environmental stability over 3000 h under RH of 40%. We believe this study provides an efficient alternative of improving the carrier conducting capability for charge transport layers and their optoelectronic devices, from the viewpoint of p–n homojunction engineering.

## Experimental Section

### Materials

Unless stated otherwise, all materials were purchased from Sigma-Aldrich without further purification. Fluorine-doped tin oxide (FTO) coated glass substrates (around 1.5 cm × 1.5 cm) with partial etching were purchased from OPV•Tech. Spiro-OMeTAD (2,2′,7,7′-tetrakis[N,N-di(4-methoxyphenyl)amino]-9,9′-spirobifuorene, ≥ 99.8% purity), 4-tert-butylpyridine (tBP, ≥ 99.9% purity), and lithium-bis (trifluoromethanesulfonyl) imide (Li-TFSI, ≥ 99.9% purity) were purchased from Xi’an Polymer Light Technology Corp.

### Device Fabrication

#### ***Preparation of Ligand-Free Ti***_***0.936***_***O***_***2***_*** Nanocrystals in Desired Solvents***

The initial anatase Ti_0.936_O_2_ powder was synthesized using solvothermal treatment of tetrabutyl titanate in an ethanol-glycerol mixture and then thermal calcination according to precious studies [13]. A certain amount of Ti_0.936_O_2_ powder (2 mg) was then transferred into a vessel with 10 mL deionized water (0.2 mg mL^−1^), subsequently subjected to a non-focusing nanosecond pulsed laser irradiation (Quantel, repetition rate: 10 Hz, pulse width: 8 ns) with a wavelength of 355 nm, along with continuous ultrasonic treatment to obtain the colloid concentration of 0.2 mg mL^−1^. Subsequently, the colloids with 0.2 mg mL^−1^ concentration were diluted to 0.1 and 0.05 mg mL^−1^, respectively, which further suffer from laser irradiation to keep the colloids homogeneous. The tailored laser fluence ranges from 0 to 300 mJ pulse^−1^ cm^−2^ and the concentration of raw Ti_0.936_O_2_ powder ranges from 0.05 to 0.2 mg mL^−1^ at fixed irradiation time of 3 min and cooling temperature of − 20 °C.

#### Preparation of Compact ETLs

The etched FTO substrates were washed successively in detergent, deionized water, acetone and ethanol under continuous sonication, then dried with N_2_ flow employing a compressed nitrogen gun, and then treated under oxygen plasma for 10 min to remove organic residues on FTO substrates.

For the preparation of target ETL: an approximately 50 nm thick TiO_2_ compact layer deposited on clean FTO substrate is prepared adopting in situ chemical bath co-deposition by adding different volume of Ti_0.936_O_2_ colloid solutions (3%, 6%, and 9% volume ratio to TiCl_4_ precursor abbreviated as 3%-Target TiO_2_, 6%-Target TiO_2_, 9%-Target TiO_2_) to TiCl_4_ aqueous solution (2.25: 100 volume ratio of TiCl_4_: H_2_O), along with FTO substrates suffering from thermal treatment at 70 °C for 1 h, and then anneal at 150 °C for 1 h.

For the preparation of pristine ETL: identical TiCl_4_ aqueous solution without adding Ti_0.936_O_2_ colloid solutions, along with FTO substrates are subjected to similar thermal processing mentioned above.

#### Fabrication of Perovskite Solar Cells

CsFAMA type perovskite: CsI (0.0625 M), FAI (1.0125 M), PbI_2_ (1.075 M), MABr (0.175 M) and PbBr_2_ (0.175 M) is dissolved in a mixture of DMF: DMSO (4:1 v/v) with a successive stir at 55 °C for 2 h to prepare precursor solution at a concentration of 1.25 M. The spin-coating process was performed in a nitrogen glove box. The as-prepared precursor (30 μL) was dropped onto the TiO_2_/FTO substrate followed by a consecutive two-step spin-coating process at 2000 and 4000 rpm for 10 and 30 s, respectively. During the second spin-coating step, 200 μL anhydrous chlorobenzene (CB) was immediately poured on the substrate 10 s prior to the end of the program. Subsequently, the intermediate phase film is heated on a hotplate at 100 °C for 1 h.

FAPbI_3_ type perovskite: FAI (1.80 M), PbI_2_ (1.80 M), FAHCOO (0.12 M), and MACl (0.52 M) are dissolved in a mixture of DMF: DMSO (volume ratio = 4:1) at 55 °C for 2 h to prepare the perovskite precursor (1.80 M). The as-prepared precursor (30 μL) was dropped onto the TiO_2_/FTO substrate followed by a one-step spin-coating at 6500 rpm for 60 s. During spin coating, 200 μL chlorobenzene (CB) is immediately poured on the substrate 20 s prior to the end of the program. Subsequently, the intermediate phase film is heated on a hotplate at 150 °C for 10 min.

In addition, for FAPbI_3_ type perovskite, 3 mg ml^−1^ phenethylammonium iodide (PEAI) was deposited on the perovskite/TiO_2_/FTO substrate via a one-step spin-coating process at 4000 rpm for 30 s, followed by deposition of Spiro-OMeTAD.

Spiro-OMeTAD solution was prepared by dissolving 72.3 mg Spiro-OMeTAD, 29 μL 4-*tert*-butylpyridine (tBP) and 18 μL lithium-bis (trifluoromethanesulfonyl) imide (Li-TFSI, a stock solution of 520 mg mL^−1^ in acetonitrile) into 1 mL chlorobenzene. And then 30 μL solution was spin-coated on the perovskite/TiO_2_/FTO substrate at 6000 rpm for 30 s. Finally, the Au electrode (80 nm) was deposited on the top of devices by thermal evaporation using a shadow mask. Each electrode of devices exhibits the area of 0.05 cm^2^, for which the effective area would be corrected by optical microscopy.

### Characterization

The scanning electron microscopy (SEM) images were obtained using a field emission SEM (FEI Nova). Atomic force microscope (AFM) was carried out using a Bruker Dimension Icon. High-resolution transmission electron microscopy (HRTEM) was conducted employing an FEI Tecnai F30 transmission electron microscope at 300 kV, equipped with an Oxford Instruments EDS detector and a high angle annular dark field (HAADF) STEM detector. The Raman spectra were recorded by a Raman microscope at an excitation laser wavelength of 532 nm (Renishaw). The X-ray diffraction (XRD) patterns were recorded on a X’pert PRO (PANalytical) adopting a Cu Kα (*λ* = 0.15406 nm) as the X-ray source. The absorption was characterized by the ultraviolet–visible (UV–vis) spectrophotometer (Perkin-Elmer Lambda 35 UV–vis-NIR). The steady-state photoluminescence (PL) and time-resolved photoluminescence (TRPL) spectra were recorded by a pulse laser excitation source at the wavelength of 470 nm (Horiba FluorologFL-3). The electrical impedance spectroscopy (EIS) was characterized applying a bias of 0.8 V in the dark in a frequency range from 1 MHz to 0.1 Hz (CHI660E). For Mott-Schottky analysis, capacitance–voltage measurements were performed at a frequency of 1 kHz (CHI660E). X-ray photoelectron spectroscopy (XPS) measurements were conducted on an Axis Supra (Kratos). Ultraviolet photoelectron spectroscopy (UPS) was characterized by a VG Scienta R4000 analyzer and the HeI (21.22 eV) emission line employed for excitation at a bias of − 5 V. The contact angles measurements were conducted by a data physics OCA-20 contact-angle system at ambient air. Temperature dependent admittance spectroscopy (TAS) was performed on a precision impedance analyzer at various temperatures (*T* = 210–320 K) in the dark. A Keithley 2400 source meter was used to record the *J*–*V* curves and maximum power point tracking under simulated AM 1.5G illumination (100 mW cm^−2^) produced by a xenon-lamp-based solar simulator (Oriel 67005, 150 W Solar Simulator), which was calibrated with a monocrystalline silicon reference cell (Hamamatsu S1133). The devices were measured both in reverse scan (+ 1.2–− 0.1 V) and forward scan (− 0.1– + 1.2 V) with a scanning rate of 0.2 V s^−1^. The EQE was conducted by employing a Enlitech EQE measurement system (QE-R3011). A Keithley 2400 source was used to measure the dark *I*–*V* characterization of the electron-only devices for calculating the defect density using SCLC model.

### Statistical Analysis

All quantitative values are shown as means ± standard deviation. All quantitative experiments were carried out using at least three replicates for each group. The statistical analysis was conducted by the *t* test, and a *p* value of less than 0.05 was considered as statistical significance. The error bars correspond to the standard deviation of data points from individual samples.

## Results and Discussion

### Laser Embedding of p-Type Ti_0.936_O_2_ Nanocrystals in TiO_2_ ETLs

**Fig. 1:**
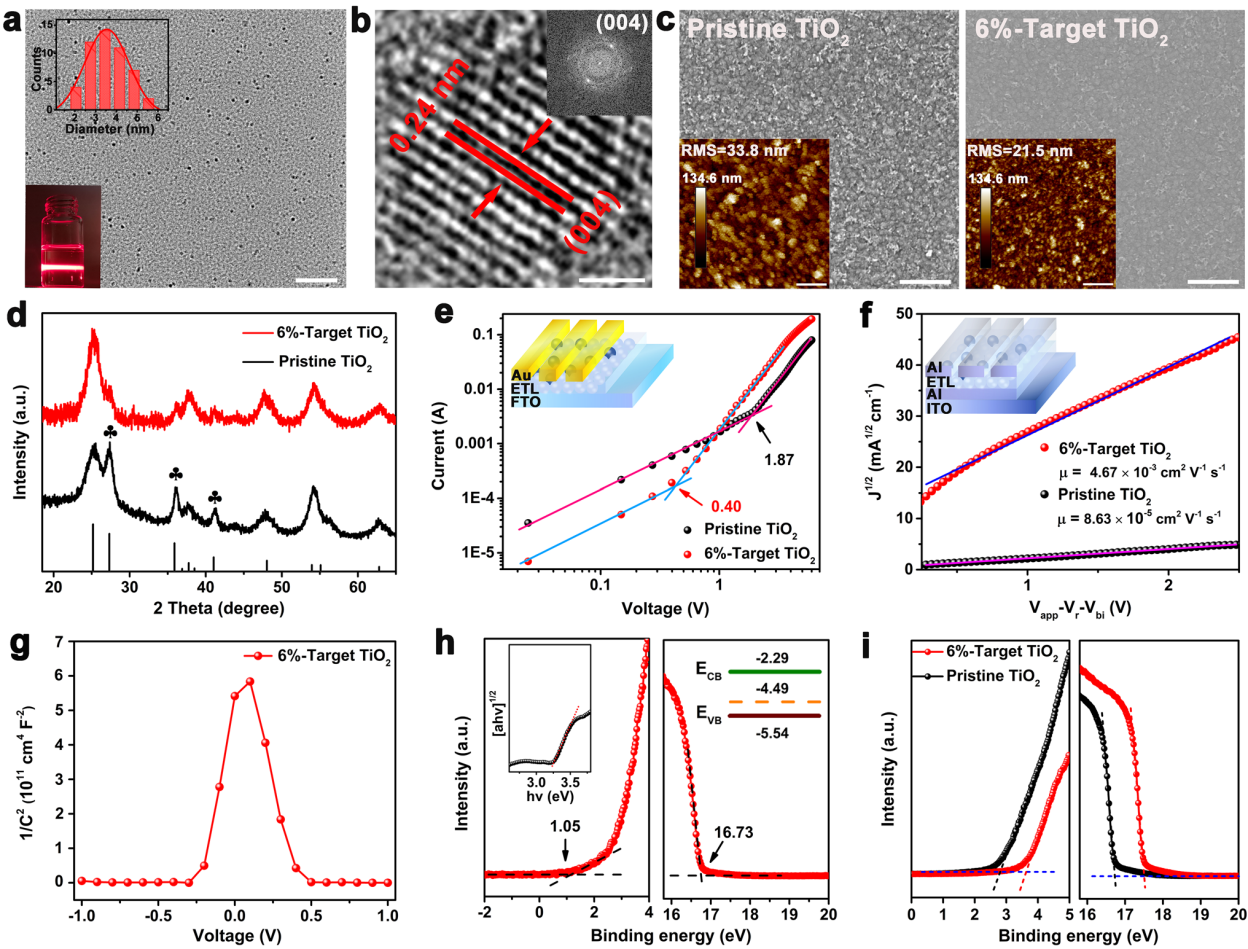
**a** TEM image of Ti_0.936_O_2_ nanocrystals (inset: nanocrystals size distribution diagram and Mie-scattering image of colloids). **b** HRTEM and corresponding FFT of Ti_0.936_O_2_ nanocrystals. **c** SEM images (inset: AFM images) of pristine TiO_2_ and 6%-target TiO_2_ films. **d** XRD patterns of different ETLs. **e** Dark *I*–*V* measurement of the electron-only devices (inset) displaying *V*_TFL_ kink point related to the trap density. **f** Electron mobilities of different ETLs using the SCLC model, the inset shows the device structure of ITO/Al/ETLs/Al. **g** Mott-Schottky plots of Ti_0.936_O_2_@TiO_2_ matrix. **h** UPS results of Ti_0.936_O_2_ nanocrystals (inset: band gap and energy level of Ti_0.936_O_2_ nanocrystals). **i** Different TiO_2_ films with UPS Fermi edge (left) and the cut-off energy (right). Scale bar: **a** 50 nm; **b** 1 nm; **c** 1 μm (inset: 1 μm)

Laser manufacture of size-tailored Ti_0.936_O_2_ nanocrystals by irradiation of their raw sub-micrometer counterpart in liquid and subsequent embedding in the TiO_2_ matrix through the chemical bath deposition method are shown in Figs. S1 and S2. Subsequent to the optimization of laser fluence and concentration of laser process (Fig. S3), the transparent Ti_0.936_O_2_ colloid solution with clear Tyndall scattering is obtained with well-dispersed nanocrystals with an average diameter of 3.5 nm (Fig. 1a). The crystal structure of the as-prepared Ti_0.936_O_2_ nanocrystals, which was determined by HRTEM and corresponding Fast Fourier transform (FFT), represents the lattice spacing of 0.24 nm that corresponds to the typical plane (004) of Ti_0.936_O_2_ (Fig. 1b), further confirmed by their identical Raman spectroscopy (Fig. S4). These results indicate that Ti_0.936_O_2_ nanocrystals well inherit the properties of their bulk counterpart. In addition, the elements mapping extracted from TEM-energy-dispersive spectroscopy (TEM-EDS) suggests homogeneous distribution of all elements throughout the entire Ti_0.936_O_2_ nanoparticles without any segregation (Fig. S5). Furthermore, the XPS demonstrate stable surface composition and chemical state of the Ti_0.936_O_2_ during laser irradiation (Fig. S6) [13]. This is consistent with the recent work that reflects unchanged Ti-vacancy of laser-processed Ti_0.936_O_2_ characterized by electron paramagnetic resonance (EPR) spectroscopy [14].

Subsequently, the deposition of Ti_0.936_O_2_@TiO_2_ ETL was conducted adopting a facile one-step chemical bath co-deposition, where laser-generated sub-5 nm Ti_0.936_O_2_ nanocrystals could be in situ embedded in the TiO_2_ matrix (Fig. S7). In brief, different contents (3%, 6%, and 9% volume ratio to TiCl_4_ precursor denoted as 3%-target TiO_2_, 6%-target TiO_2_, 9%-target TiO_2_) of Ti_0.936_O_2_ colloids with concentration of 0.1 mg mL^−1^ were incorporated into TiCl_4_ solution to fabricate the TiO_2_ composite ETLs. Scanning electron microscopy (SEM) and atomic force microscopy (AFM) were performed to evaluate the surface morphologies of corresponding TiO_2_ films. The results reveal that the 6%-target TiO_2_ film exhibits smoother and flatter surface with reduced roughness from 33.8 to 21.5 nm in comparison with the pristine film, as shown in Figs. 1c and S8. In order to further explore the influence of embedding Ti_0.936_O_2_ on crystallization kinetics of TiO_2_ ETLs, the settled TiO_2_ powders after chemical bath co-deposition were collected for systematic XRD analysis. As shown in Fig. 1d, the initial TiO_2_ ETL consists of polymorphic phases of rutile and anatase, while the rutile phase in the ETLs is significantly reduced after the embedding of Ti_0.936_O_2_. It should be noted that the fast nucleation rate is more inclined to form the anatase phase at the initial stage of TiCl_4_ hydrolysis, whereas the slow nucleation rate is conducive to the directional arrangement of aggregates, resulting in the generation of a more stable rutile phase [15]. It could thus be deduced that the addition of Ti_0.936_O_2_ favors the rapid nucleation, which enables not only the formation of smaller TiO_2_ grains for their compact deposition, but the inhibition of rutile phase and anatase–rutile hetero-phase junction for reducing the unwanted photocatalytic degradation of PSCs under continuous light soaking [16, 17].

In order to investigate the effects of the Ti_0.936_O_2_ nanocrystals on the optical and electronic properties of the TiO_2_ ETLs, the optical bandgap of Ti_0.936_O_2_@TiO_2_ films were first evaluated by ultraviolet–visible (UV–vis) absorption spectra and corresponding Tauc plots. The results show that the embedding of Ti_0.936_O_2_ enables slight increase in the bandgap of the TiO_2_ ETLs from 3.20 to 3.22 eV, with insignificant change on the optical transmittance, as shown in Figs. S9 and S10. To evaluate the electronic properties, the defect density (*N*_t_) and the electron mobility (*µ*) of the TiO_2_ ETLs were successively examined by the space charge-limited current (SCLC) method. The result shows that the embedding of Ti_0.936_O_2_ results in the reduction of *N*_*t*_ from initial 6.48 × 10^16^–1.39 × 10^16^ cm^−3^ (Fig. 1e and Table S1), which may be due to the improved crystallization kinetics of TiO_2_ and the high-quality ETLs. Moreover, the *µ* is found to be boosted by two orders of magnitude from pristine 8.63 × 10^–5^–4.67 × 10^–3^ cm^2^ V^−1^ s^−1^ for the 6%-target TiO_2_ ETLs (Fig. 1f), which is consistent with the conductivity (*σ*) result that indicates higher *σ* for 6%-target TiO_2_ due to a large slope (Fig. S11). The improved electronic properties are mainly attributed to the construction of Ti_0.936_O_2_@TiO_2_ p–n homojunction, which is evidenced by an inverted “V-shape” with typical p–n junction feature from the Mott-Schottky plot shown in Fig. 1g [18]. To confirm the p-type characteristic of the Ti_0.936_O_2_, the ultraviolet photoelectron spectroscopy (UPS, Fig. 1h) was used to check its electronic structure of the Ti_0.936_O_2_. Based on the optical bandgap (3.25 eV) (inset in Fig. 1h), the corresponding Fermi energy level, the conduction band energy level and the valence band energy level are calculated to be − 4.49, − 2.29, and − 5.54 eV, respectively, which identifies the p-type semiconductor feature of the Ti_0.936_O_2_ nanocrystals. Such p–n construction greatly accelerates the carrier transport at both the surfaces and the boundaries of TiO_2_ particles to restrain carrier loss owing to the increase of the depletion width [18, 19]. It is also found that the embedded p-n homojunction is helpful to improve electronic structure of TiO_2_ ETLs with upward-shifted energy level, enabling a better energy level alignment with top perovskite active layer to lower the interfacial electron barrier (Figs. 1i and S12) [9]. Detailly, the UPS characterization of laser-processed Ti_0.936_O_2_ nanocrystals strongly confirms their p-type semiconductor characteristic (Fig. 1h), which were embedded into the n-type TiO_2_ matrix to form a p–n junction by generating a uniform Fermi level (Fig. S12b). It is worth noting that the formed p–n homojunctions between Ti_0.936_O_2_ and TiO_2_ could create numerous localized built-in electric fields with a direction from n-type TiO_2_ to p-type Ti_0.936_O_2_, as shown in Scheme 1 and Fig. S12b, which enables not only the effective promotion of carrier transport at both the surfaces and boundaries of TiO_2_ matrix due to the expansion of the depletion width [19], but also the oriented transport of photo-generated charge carriers, which favors for the boosted electron mobility (Scheme 1) [10].

### Effect of Ti_0.936_O_2_@TiO_2_ on the Top Perovskite Films

**Fig. 2:**
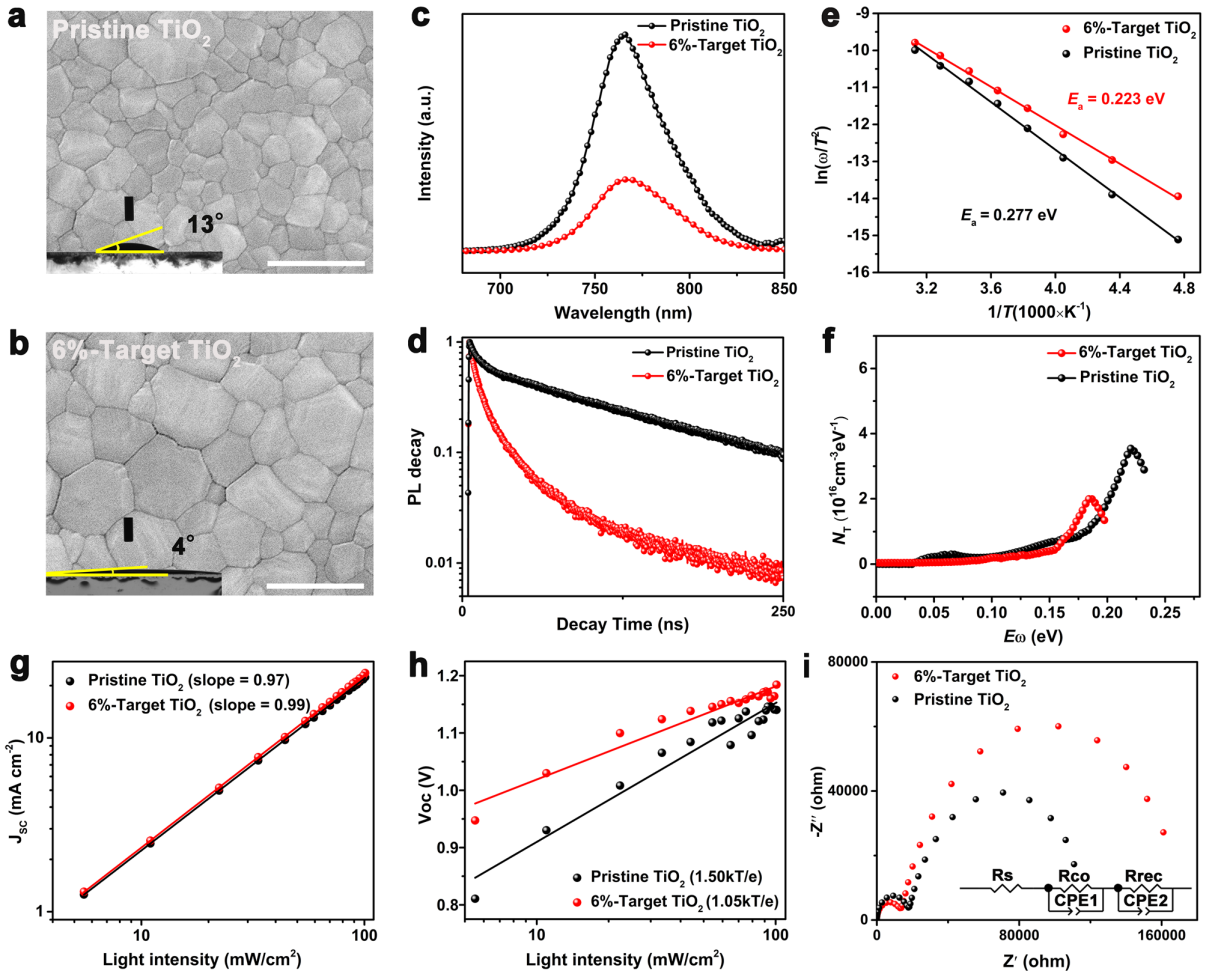
SEM top-view images of perovskite films based on **a** pristine TiO_2_ and **b** 6%-target TiO_2_ films (inset: contact angles of different ETLs dropped by perovskite precursor). Steady-state **c** and time-resolved **d** PL spectra of CsFAMA perovskite films spin-coated on different TiO_2_ layers. **e** Arrhenius plots of the characteristic transition frequencies. **f** Trap state density (*N*_T_) of the perovskite photovoltaics measured at 300 K. Dependence of **g** *J*_sc_ and **h** *V*_OC_ on the irradiation intensity of the devices based on different TiO_2_ ETLs. **i** Nyquist plots of the devices based on different ETLs measured in the dark at a bias of 0.8 V. Scale bar: **a** 500 nm; **b** 500 nm

The surface morphologies of the mixed-cation perovskite (Cs_0.05_(FA_0.85_MA_0.15_)_0.95_PbI_2.55_Br_0.45_, CsFAMA) grown on different TiO_2_ ETLs were intentionally evaluated by the SEM and AFM characterizations. The results show obviously larger-grain size and smoother surface of target perovskite films compared with those of pristine ones (Figs. 2a, b and S13). Such surface morphology can trigger the reduction of Gibbs free energy for heterogeneous nucleation of perovskite precursor due to the sharply decreased contact angle from pristine 13°–4°, for Ti_0.936_O_2_@TiO_2_ ETLs (see insets in Fig. 2a, b), thus contributing to the high-quality and large-grain perovskite films [20]. Cross-sectional SEM and XRD patterns demonstrate the large grains throughout the entire thickness of the perovskite film deposited on Ti_0.936_O_2_@TiO_2_ ETLs (Figs. S14 and S15), owing probably to the enhanced crystallization kinetics during their grain growth [6]. Furthermore, the steady-state and time-resolved PL spectra were employed to investigate the effects of Ti_0.936_O_2_ nanocrystals on the carrier dynamics between the perovskite layer and the ETLs. As shown in Fig. 2c, the CsFAMA perovskite films based on the 6%-target TiO_2_ present a more prominent PL quenching with a twofold decrease of the PL intensity compared with the control films, demonstrating more efficient electron transfer between the perovskite layer and the ETLs. Similarly, time-resolved PL (TRPL) results (Fig. 2d, Table S2) exhibit that the average carrier lifetimes are calculated to be 130.63–37.38 ns for the control and the target, respectively, indicating the significant reduction of the carrier lifetime and thereby the enhanced electron extraction at the buried interface [16].

In order to further explore the impact of Ti_0.936_O_2_@TiO_2_ ETLs on modulating defect states in top perovskite films, temperature dependent admittance spectroscopy (TAS) was accordingly employed to quantitatively estimate both the energy level and the distribution of trap states (Note S1, Fig. S16) [21]. As shown in Fig. 2e, the defect activation energies (*E*_a_) of different films are extracted from the Arrhenius plots of the characteristic transition frequencies obtained from the corresponding capacitance-frequency curves under different temperatures (Fig. S16a, b), and are calculated to be 0.277 and 0.223 eV for the control and target films respectively. Figure 2f exhibits the energy level and the density of trap states of different perovskite films, demonstrating that Ti_0.936_O_2_@TiO_2_ ETLs effectively reduce the energy level of trap states from pristine 0.22–0.18 eV, as well as their density of states from pristine 3.52 × 10^16^–2.03 × 10^16^ cm^−3^. Subsequently, the short-circuit current density (*J*_sc_) and the open-circuit voltage (*V*_oc_) at variable light intensities were measured to gain in-depth understanding of the carrier recombination kinetics in perovskite films. As shown in Fig. 2g, the curves of dependence of *J*_sc_ on the irradiation intensity represent similar slopes close to 1, revealing negligible bimolecular recombination within all films [22]. Figure 2h depicts *V*_oc_ versus light intensity in which the fitted slopes significantly decrease from pristine 1.50*–*1.05*kT*/*e* for target films, indicating the effectively suppressive trap-assisted recombination that facilitates leakage current (Fig. S17) [22], which is also in good agreement with that of the TAS in Fig. 2f. The EIS was used to reflect interfacial charge transfer capability between the perovskite layer and ETLs. As shown in Fig. 2i, the contact resistance (*R*_co_) decreases from pristine 16,544–12,300 Ω and the recombination resistance (*R*_rec_) increases from pristine 1.05 × 10^5^–1.64 × 10^5^ Ω for the 6%-target TiO_2_ (Table S3). These results indicate that embedding of the Ti_0.936_O_2_ in TiO_2_ matrix results in effectively improved charge transport and suppressed charge recombination at the buried interface [16, 23, 24].

### Photovoltaic Performance and Stability of Planar PSCs Based on Ti_0.936_O_2_@TiO_2_ ETLs

**Fig. 3:**
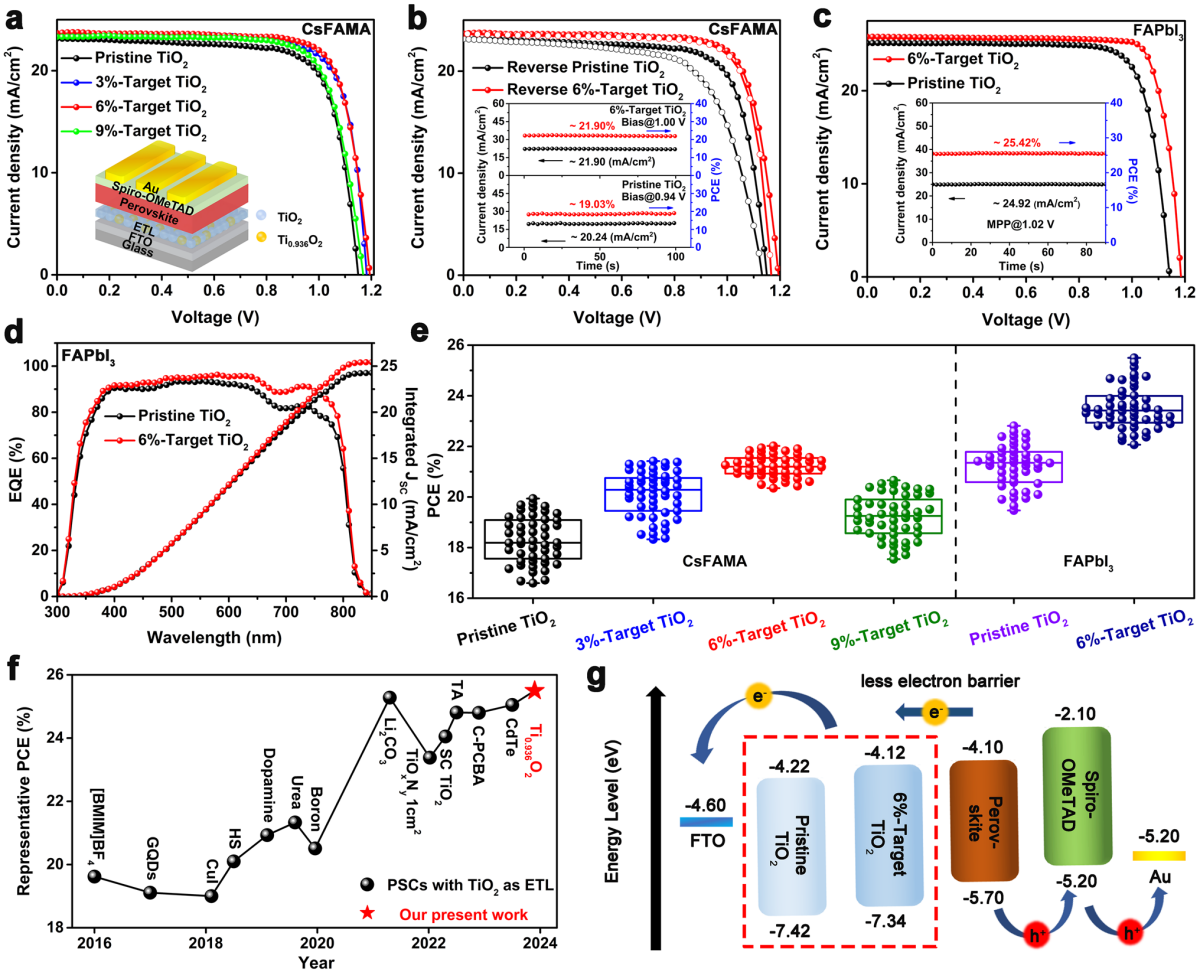
**a** *J-V* curves of CsFAMA devices with different TiO_2_ layers (inset: schematic illustration of device structure). **b** *J*–*V* plots of CsFAMA champion devices containing the pristine TiO_2_ and 6%-target TiO_2_ layers measured both in reverse scan and forward scan, the insets show stabilized power output at MPP tracking. **c** *J*–*V* curves of FAPbI_3_ devices employing different TiO_2_ layers with stabilized power output at MPP tracking. **d** EQE spectra of FAPbI_3_ champion devices upon the pristine TiO_2_ and 6%-target TiO_2_ layers, respectively. **e** PCE distribution of 50 individual CsFAMA and FAPbI_3_ devices. **f** Comparison of efficiency of PSCs employing TiO_2_ as ETLs. **g** Energy level diagram for each component of devices upon different TiO_2_ layers. The energy level structures of Spiro-OMeTAD and Au refer to the literature [23, 24]

The regular planar PSCs with the configuration of FTO substrate/Ti_0.936_O_2_@TiO_2_/perovskite/Spiro-OMeTAD/Au were fabricated to further evaluate the effect of the embedding of Ti_0.936_O_2_ on photovoltaic performance (See schematic illustration of fabrication process in Fig. S18). Figure 3a shows the current density–voltage (*J*–*V*) curves of different CsFAMA champion devices measured under illumination of 100 mW cm^−2^ (AM 1.5G) and the corresponding photovoltaic parameters are listed in Table 1. The device based on 6%-target TiO_2_ ETLs exhibits a PCE of 22.02% with a *V*_oc_ of 1.194 V, a *J*_sc_ of 23.72 mA cm^−2^, and a fill factor (FF) of 77.75%, higher than those of devices based on pristine TiO_2_ (19.94%), 3%-target TiO_2_ (21.42%), and 9%-target TiO_2_ (20.66%). In addition, negligible hysteresis is shown in target champion devices with a stabilized power output of 21.90% close to maximum efficiency (Fig. 3b), evidenced by reduction of hysteresis factor from pristine 13.1%–1.5% for the 6%-target TiO_2_-based devices (Table S4). To verify the universality of Ti_0.936_O_2_@TiO_2_ ETLs in enhancing carrier dynamics at buried interface, α-phase formamidinium lead iodide (FAPbI_3_)-based devices were further constructed to pursue higher photovoltaic performance. It is worth noting that Ti_0.936_O_2_@TiO_2_ ETLs could encouragingly initiate the fabrication of highly crystalline FAPbI_3_ perovskite with optimized morphology, and enable enhanced carrier dynamics at the buried interface (Figs. S19, S20, S21. S22, S23, Table S5). Owing to these merits, the target FAPbI_3_ PSC upon a bandgap of 1.53 eV (Fig. S24) achieved the champion PCE up to 25.50% with a *V*_oc_ of 1.185 V, a *J*_sc_ of 25.79 mA cm^−2^, and a *FF* of 83.45%, and highly stabilized PCE of 25.42%, far exceeding that of the control (22.81%), as shown in Fig. 3c and Table 1. Figure 3d further exhibits external quantum efficiency (EQE) spectra, in which enhanced spectral response of target devices in entire range is due to the construction of high-quality and large-grain perovskite films deposited on Ti_0.936_O_2_@TiO_2_ ETLs [25], enabling an increment of integrated *J*_sc_ from 24.27 to 25.43 mA cm^−2^, matching well with the *J*–*V* results. As shown in Fig. 3e, efficiency distribution histogram of 50 individual PSCs indicates the improved reproducibility of the target devices, demonstrated by enhanced average PCEs from 18.28% to 21.22% and from 21.24% to 23.52% that successively corresponds to CsFAMA and FAPbI_3_ PSCs, along with synchronous improvement of *V*_oc_, *J*_sc_ and *FF* (Figs. S25 and S26). It is worth noting that champion efficiency of 25.50% in present work ranks among the top in records of PSCs based on TiO_2_ ETLs (Fig. 3f, Table S6). Such significant enhancement in efficiency is partially attributed to the optimized interface band-alignment induced by the embedding of Ti_0.936_O_2_ (Fig. 3g), thereby favoring the less charge accumulation at the interface between perovskite and ETL and the increment of voltage output, evidenced by the UPS analyses and capacitance–voltage measurements (Figs. S16c and S27) [26–28].

**Table 1:** Photovoltaic parameters of the CsFAMA and FAPbI_3_ type PSCs upon different TiO_2_ ETLs

Sample	Content of Ti_0.936_O_2_ (%)	*V*_OC_ (V)	*J*_SC_ (mA cm^−2^)	FF (%)	PCE (%)	Average PCE (%)
Pristine TiO_2_	0	1.149	23.16	74.93	19.94	18.28
3%-Target TiO_2_	3	1.181	23.50	77.18	21.42	20.13
6%-Target TiO_2_	6	1.194	23.72	77.75	22.02	21.22
9%-Target TiO_2_	9	1.169	23.32	75.80	20.66	19.24
Pristine FAPbI_3_	0	1.142	25.10	79.57	22.81	21.24
Target FAPbI_3_	6	1.185	25.79	83.45	25.50	23.52

**Fig. 4:**
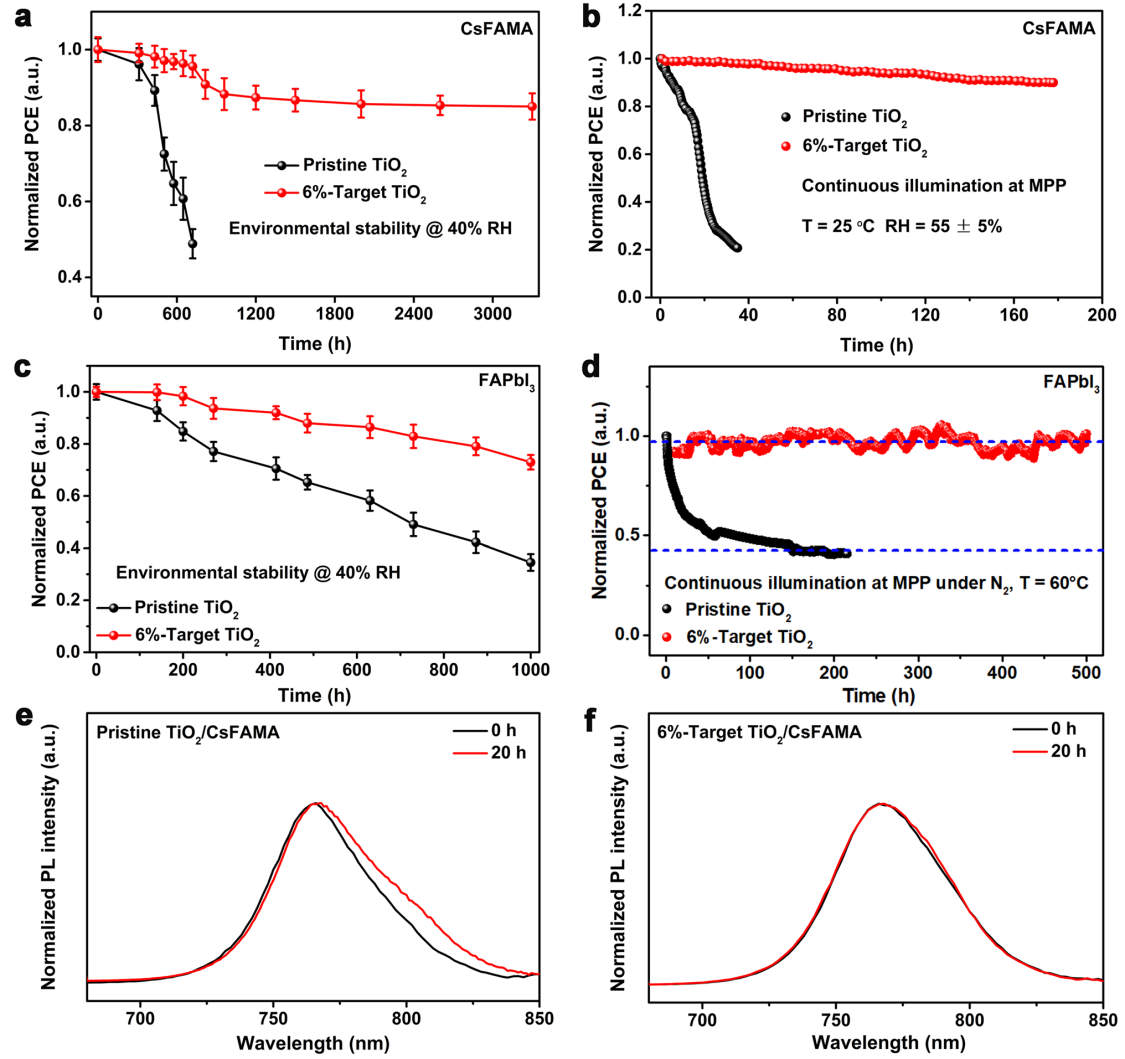
**a** Humidity stability and **b** operational stability of different CsFAMA-based devices. **c** Humidity stability and **d** operational stability of different FAPbI_3_-based devices. Normalized PL spectra of perovskite films grown on **e** pristine TiO_2_ and** f** 6%-target TiO_2_ layers under continuous 254 nm UV irradiation (50 mW cm^−2^) for 20 h. The error bars represent the standard deviation for 20 devices

Adopting the p–n homojunction embedding strategy has been demonstrated to be greatly effective for the construction of high-performance device, while its influence on the long-term stability of devices would be further investigated. Figure 4a shows the humidity stability of different CsFAMA PSCs without encapsulation stored in ambient air with relative humidity (RH) of 40% in the dark. The result indicates that the target devices exhibit superior humidity stability, maintaining 85% of initial PCE for 3300 h in comparison with that of control devices (approximately 50% for 700 h). The operational stability of target devices also shows great improvement, retaining 93% of initial PCE over 170 h in contrast with that of control devices (approximately 21% for 35 h), which was measured using maximum power point (MPP) tracking under full-sun illumination in ambient air with RH of 55 ± 5%, as well as excellent thermal stability (Figs. 4b and S28). We further check the environmental stability of various FAPbI_3_-based devices, due to the ease with which FAPbI_3_ perovskite could arouse its spontaneous phase transition from α- to δ-FAPbI_3_ under ambient conditions [29]. For the humidity stability of FAPbI_3_ PSCs (RH of 40%, Fig. 4c), the control devices continuously degrade by more than 60% of their initial PCE for 1000 h, whereas the target devices could retain 73% of their initial value at the same time. For the operational stability of FAPbI_3_-based devices under MPP tracking at 60 °C under full-sun illumination in inner atmosphere, the enhanced operational stability in target FAPbI_3_ device is demonstrated by less than 5% degradation of its initial PCE over 500 h, compared with that of control devices (over 50% after 200 h, Fig. 4d). The PL characterization was further carried out to check the stability of perovskite films under continuous UV irradiation. As shown in Fig. 4e, f, there is a red shift of about 3 nm in the PL peak of pristine perovskite film under UV irradiation of 20 h, while target perovskite films on Ti_0.936_O_2_@TiO_2_ ETLs exhibit a negligible shift, demonstrating suppressive decomposition of perovskite film due to significantly reduced anatase–rutile phase junctions that trigger the photocatalytic property of TiO_2_ [30], which accounts for improvement of light-illumination stability of target device.

It is encouragingly found that the Ti_0.936_O_2_@TiO_2_ ETLs play an important role in the construction of highly efficient and stable PSCs through multiple pathways: (i) the formation of the p–n homojunctions between Ti_0.936_O_2_ and TiO_2_ could not only accelerate the electron transport at both the surfaces and the boundaries of TiO_2_ matrix due to the increase in depletion width [19], but also significantly enhance crystal quality of the ETLs, resulting in enhanced conduction capability and boosted electron mobility by two orders of magnitude for TiO_2_ ETLs; (ii) such p–n homojunction enables upward-shifted Fermi level of TiO_2_ that favors better energy level alignment with the perovskite, leading thus to reduction in voltage loss and promotion of electron extraction at the buried interface [31]; (iii) embedding Ti_0.936_O_2_ could greatly improve not only crystallization process of TiO_2_ for the inhibition of rutile phase that results in light-induced instability (Fig. S29), but surface wettability of TiO_2_ that initiates rapid nucleation of top precursor for the fabrication of highly crystalline and large-grain perovskite films, which effectively prevent moisture from penetrating at grain boundaries to retard degradation of perovskite and enhance humidity stability of PSCs. Owing to these merits, highly efficient FAPbI_3_ PSCs delivered champion PCE up to 25.50%, which ranks among the top in records of TiO_2_-based planar PSCs, as well as prominent moisture (RH of 40% for 3300 h) and light (under MPP for 500 h) stability of unencapsulated PSCs were achieved. It could be inferred from the improvement of all photovoltaic parameters that better energy level alignment, accelerated electron transport and reduced contact impedance at the buried interface principally account for the increment of *V*_oc_, *J*_sc_, and *FF*, respectively [11, 23, 24]. In addition, the significantly eliminated hysteresis phenomenon of the target device is attributed to effective suppression of the carrier transport imbalance within the device, due to the enhanced electron mobility of the ETLs by embedding Ti_0.936_O_2_ (Figs. 3b and S30, Tables S4 and S7) [32].

## Conclusions

In summary, present work has demonstrated an efficient strategy of laser embedding of the p–n homojunctions in TiO_2_ ETLs to address the issues of insufficient carrier transport at the buried interface for highly efficient and stable PSCs. The embedded p–n homojunction between Ti_0.936_O_2_ and TiO_2_ not only greatly assisted synchronous acceleration of carrier transport at both surfaces and boundaries of TiO_2_, which principally accounts for boosted electron mobility by two orders of magnitude, but modulates interfacial energy level to rapidly extract electrons that results in reduction of voltage deficit. The laser generated p-type Ti_0.936_O_2_ also exerts significant influence on the crystallization kinetics of the TiO_2_ matrix and top precursor, which significantly inhibits the rutile phase that leads to light instability and initiates the fabrication of large-grain perovskite that enhances humidity stability of PSCs, respectively. The capability of such novel Ti_0.936_O_2_@TiO_2_ ETLs is thus demonstrated by accessing efficient and stable planar FAPbI_3_ PSCs with a champion efficiency of 25.50% and robust light-induced stability over 500 h, as well as mixed-cation PSCs with a champion efficiency of 22.02% and pronounced humidity stability for 3300 h (RH of 40%). This study exploits a novel pathway of developing highly conductive charge transport layers for state-of-the-art planar PSCs.

## Supplementary Information

Below is the link to the electronic supplementary material.

Supplementary file 1 (DOCX 5942 kb)
