# Supplementary material for: TiO2 Electron Transport Layer with p–n Homojunctions for Efficient and Stable Perovskite Solar Cells
Source: Nanomicro Lett. 2024 May 3;16:191. doi: 10.1007/s40820-024-01407-3 (PMC11068719; doi:10.1007/s40820-024-01407-3)
Supplement: Supplementary file 1 — (DOCX 5942 kb) [file 40820_2024_1407_MOESM1_ESM.docx]

Supporting Information for

**TiO_2_ Electron Transport Layer with p-n Homojunctions for Efficient and Stable Perovskite Solar Cells**

Wenhao Zhao^1^, Pengfei Guo^1, 2, 4,^*, Jiahao Wu^1^, Deyou Lin^1^, Ning Jia^1^, Zhiyu Fang^1^, Chong Liu^1^, Qian Ye^1^, Jijun Zou^3^, Yuanyuan Zhou^2^ and Hongqiang Wang^1, 4,^*

^1^State Key Laboratory of Solidification Processing, Center for Nano Energy Materials, School of Materials Science and Engineering, Northwestern Polytechnical University and Shaanxi Joint Laboratory of Graphene (NPU), Xi’an 710072, P. R. China

^2^Department of Chemical and Biological Engineering, Hong Kong University of Science and Technology, Clear Water Bay, Hong Kong SAR, P. R. China

^3^Key Laboratory for Green Chemical Technology of the Ministry of Education, School of Chemical Engineering and Technology, Tianjin University, Tianjin 300072, P. R. China

^4^Chongqing Innovation Center of Northwestern Polytechnical University, Northwestern Polytechnical University, Chongqing 401135, P. R. China

*Corresponding authors. E-mail: [guopengfei@nwpu.edu.cn](mailto:guopengfei@nwpu.edu.cn) (Pengfei Guo); [hongqiang.wang@nwpu.edu.cn](mailto:hongqiang.wang@nwpu.edu.cn) (Hongqiang Wang)

**Supplementary Figures**


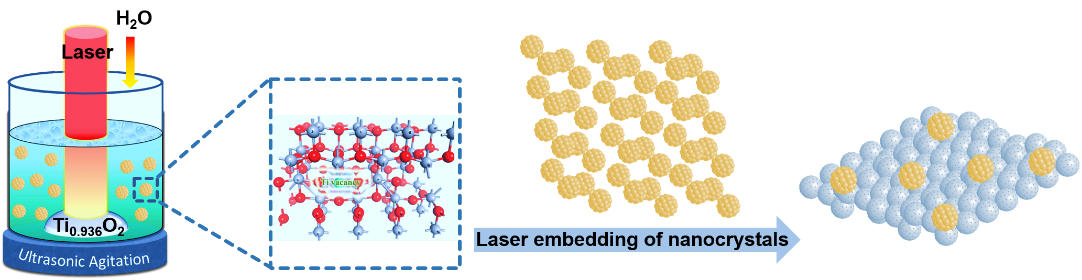


**Fig. S1** Schematic illustration of laser process of Ti_0.936_O_2_ nanocrystals and their embedding in the ETLs


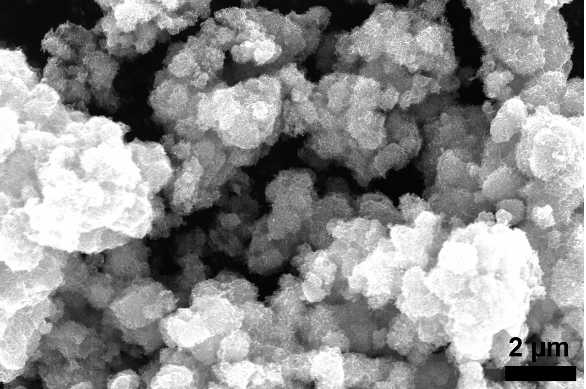


**Fig. S2** SEM image of the raw Ti_0.936_O_2_ powders before laser irradiation


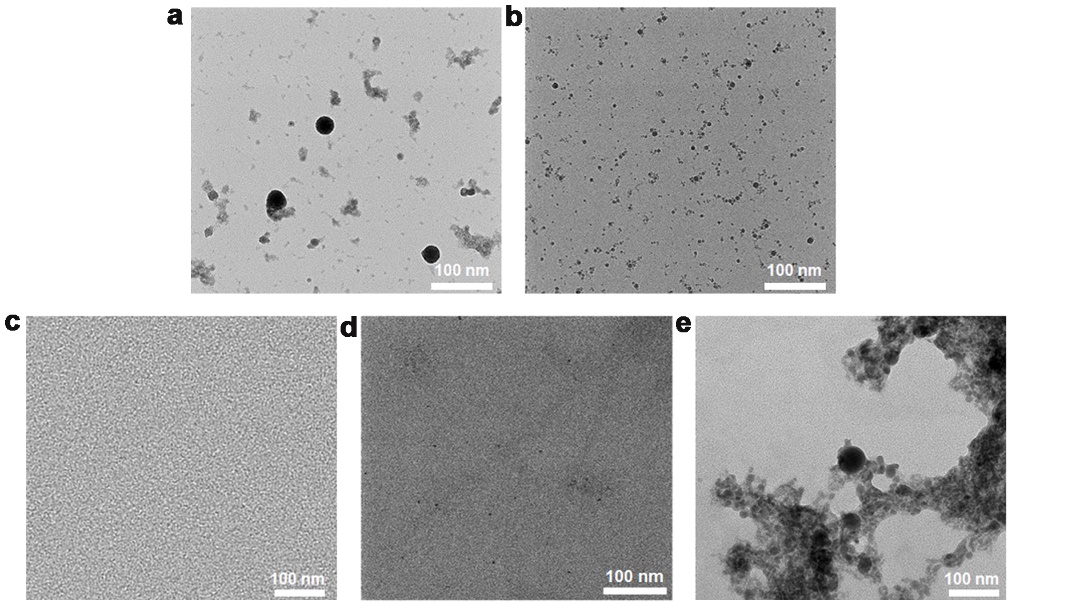


**Fig. S3** TEM images of Ti_0.936_O_2_ nanoparticles with different size under various laser fluence of **a** 100, **b** 200 and **c** 400 mJ/pulse cm^2^, respectively. TEM image of high concentration of Ti_0.936_O_2_ particles with **d** 0.05 and **e** 0.2 mg/ml

For optimization of laser fluence, Figure S3 shows TEM morphology and size evolution of Ti_0.936_O_2_ nanoparticles under different laser fluence. Normally, considering the employment of laser with the nanosecond pulse width, an increase in the laser fluence from 100 mJ/pulse cm^2^ to 200 mJ/pulse cm^2^, based on the “heat-melt-evaporation” mechanism induced by the laser in the liquid phase [S1], results in a decrease in the size of the spherical particles, as shown in Fig. S3a, b. When the laser fluence increases to 300 mJ/pulse cm^2^, Ti_0.936_O_2_ nanocrystals with a particle size below 10 nm are obtained, as shown in Figure 1a. However, if the laser fluence is further increased to 400 mJ/pulse cm^2^, it will be difficult to obtain Ti_0.936_O_2_ nanocrystals, which may be due to the strong photothermally induced evaporation effect under such conditions leading to the evaporation and further disappearance of Ti_0.936_O_2_ nanocrystals in liquid phase, as shown in Fig. S3c. Therefore, the laser fluence of 100~300 mJ/pulse cm^2^ can be used to prepare different sizes of Ti_0.936_O_2_ nanoparticles, and the optimal laser fluence is 300 mJ/pulse cm^2^.

We further explored the mechanism of the influence of target concentration on the preparation of monodisperse nanocrystals. The results shows that when the concentration of the original Ti_0.936_O_2_ particles was increased from 0.05 to 0.2 mg/ml, the laser prepared Ti_0.936_O_2_ nanoparticles would gradually change from a well-dispersed state to an agglomerated state, as shown in Fig. S3d, e. The introduction of such agglomerated Ti_0.936_O_2_ nanoparticles will keep them in the same agglomerated state in the film, which is not conducive to the preparation of smooth and flat films, and further leads to unsatisfactory interface contact and deterioration of the photoelectric performance of perovskite solar cells. Therefore, our optimal concentration of Ti_0.936_O_2_ for embedding of electron transport layer is 0.1 mg/ml.





**Fig. S4** Raman spectroscopy of Ti_0.936_O_2_ with and without laser irradiation process. Raman spectroscopy of both nanoparticles and nanocrystals of Ti_0.936_O_2_ exhibits the same Raman bands at 143 cm^-1^, 197 cm^-1^, 397 cm^-1^, 516 cm^-1^, 519 cm^-1^ (overlapping with 516 cm^-1^) and 638 cm^-1^. These peaks can be attributed to six Raman-active modes of anatase phase with the symmetries of E_g_, E_g_, B_1g_, A_1g_, B_1g_ and E_g_, respectively [S2]


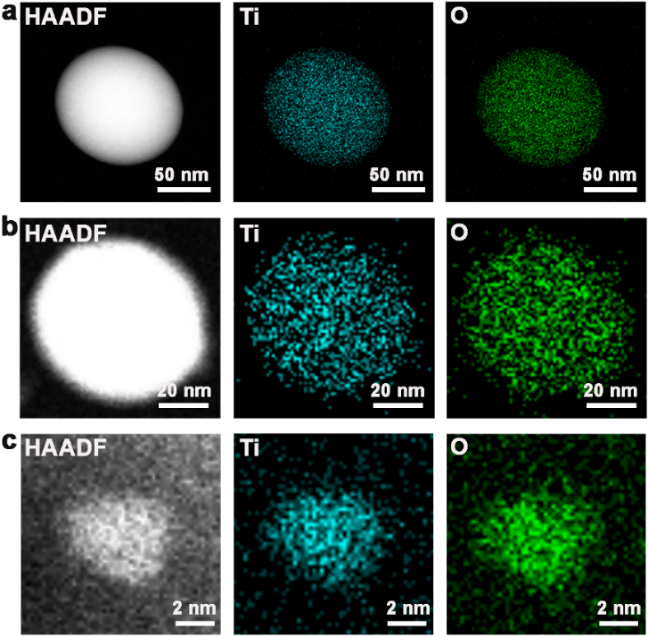


**Fig. S5** HAADF and corresponding TEM-EDS images of different size of Ti_0.936_O_2_ nanoparticles


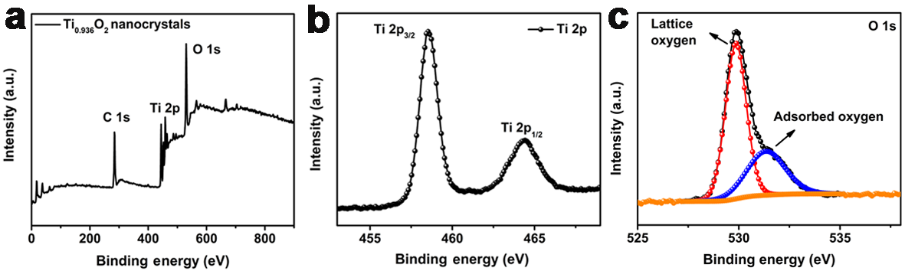


**Fig. S6** XPS spectra of Ti_0.936_O_2_ nanocrystals. Ti 2p peaks at 458.5 eV and 464.4 eV, which are attributed to the chemical states of Ti 2p_3/2_ and Ti 2p_1/2_ of Ti^4+^, respectively. The O 1s peak at 529.8 eV belongs to the Ti^4+^-O bond, and the O 1s peak at 531.4 eV belongs to the Ti-OH bond [3], which strongly confirms that Ti and O elements exist stably in Ti_0.936_O_2_ nanocrystals, and the surface composition and chemical state of Ti_0.936_O_2_ do not change significantly after laser irradiation.


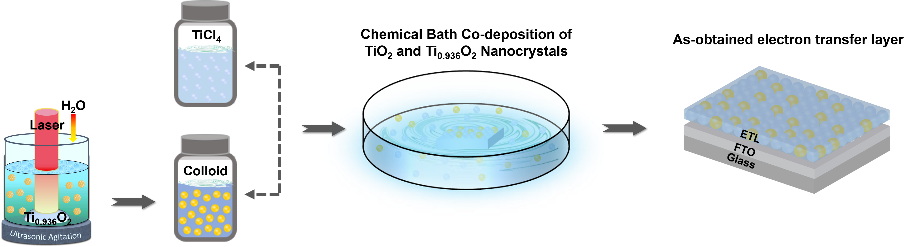


**Fig. S7** Schematic illustration of embedding of Ti_0.936_O_2_ nanocrystals in the TiO_2_ matrix


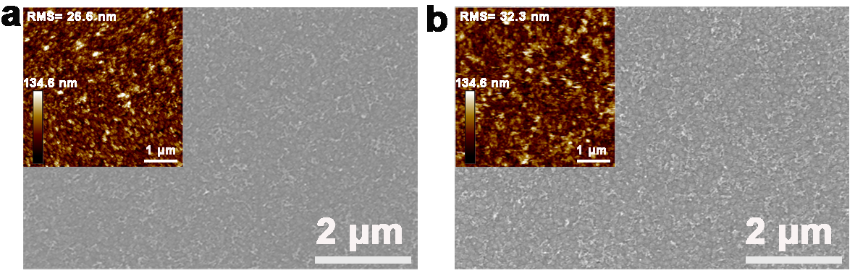


**Fig. S8** SEM images (inset: AFM images) of **a** 3%-Target TiO_2_ and **b** 9%-Target TiO_2_ films


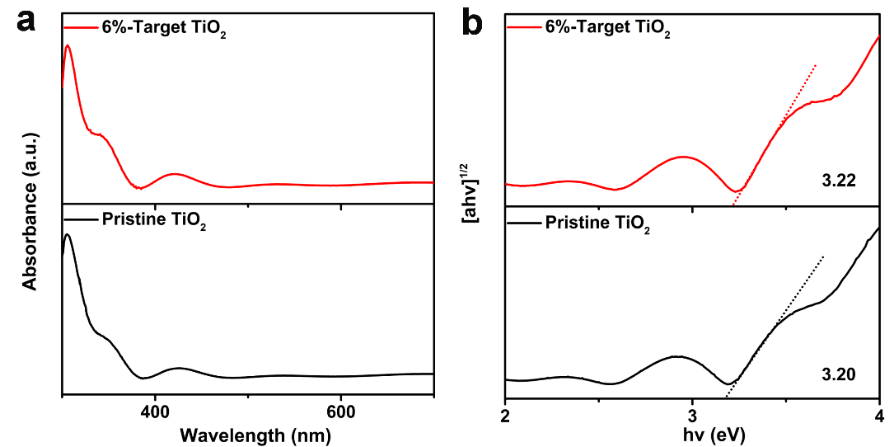


**Fig. S9** Absorption spectra **a** of different TiO_2_ films and corresponding Tauc plots **b** determining the optical bandgaps of TiO_2_ films


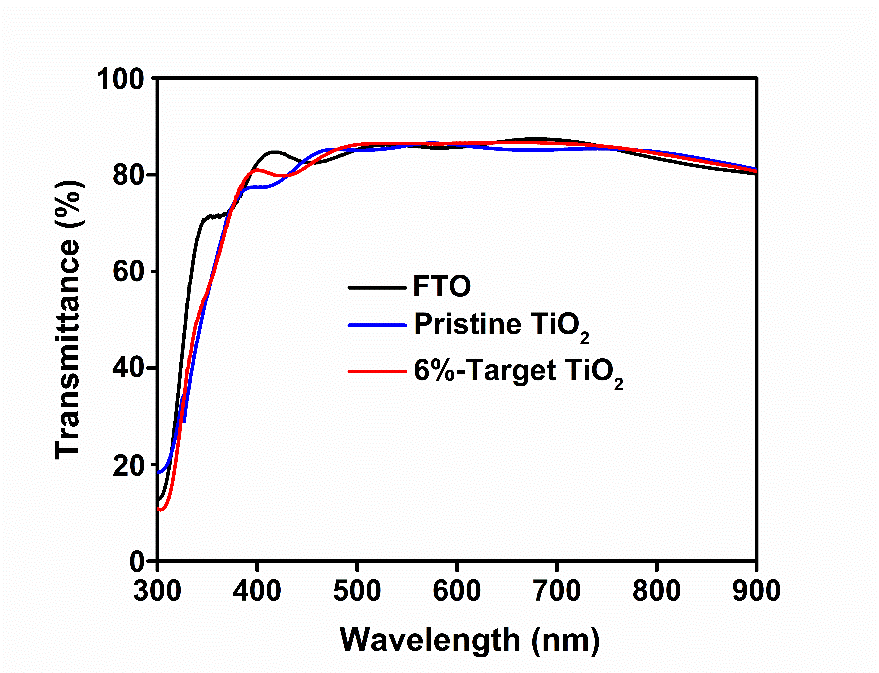


**Fig. S10** Optical transmittance spectra of FTO and different TiO_2_ films based on FTO

**Table S1** The dark *I-V* plots of different devices show *V*_TFL_ (the onset voltage of the trap-filled limit region) kink point behavior, it could be extracted to determine the *N*_t_ of various ETLs. The *N*_t_ of different ETLs was evaluated using $N_{t}=\frac{2\varepsilon_{0}\varepsilon V_{\mathrm{TFL}}}{qL^{2}}$ formula, where$\varepsilon_{0}$is the vacuum permittivity, $\varepsilon$ is the relative dielectric constant, $V_{\mathrm{TFL}}$ is the onset voltage of the trap-filled limit region, *q* is the elemental charge, and *L* is the thickness of the ETLs [S4]

| ETLs | *V*_TFL_ (V) | *N*_t_ (cm^-3^) |
| --- | --- | --- |
| Pristine TiO_2_ | 1.87 | 6.48$\times$10^16^ |
| 6%-Target TiO_2_ | 0.40 | 1.39$\times$10^16^ |





**Fig. S11** Conduction properties of pristine TiO_2_ and 6%-target TiO_2_ films measured by a device structure of FTO/ETLs/Ag


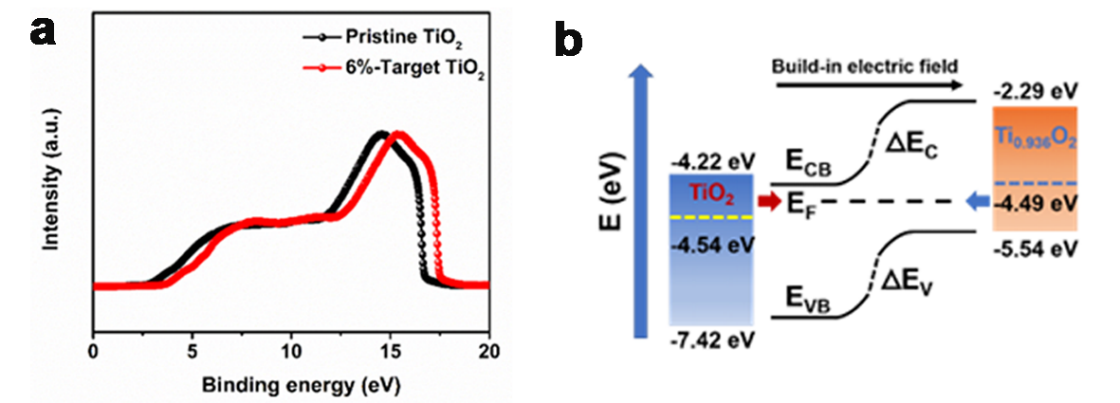


**Fig. S12** UPS spectra of different TiO_2_ films

The Fermi level (E_F_) is calculated by the equation: E_F_ = E_cut-off_ - 21.22 eV, where E_cut-off_ represents cut-off binding energy, 21.22 eV is the photon energy of excitation light. The E_cut-off_ values are extracted to be 16.68 and 17.53 eV for different TiO_2_ layers. The E_F_ values were calculated to be -4.54 and -3.69 eV for the pristine TiO_2_ and 6%-target TiO_2_ layers, respectively. Then the valence band (E_VB_) is calculated to be -7.42 and -7.34 eV for pristine TiO_2_ and 6%-target TiO_2_ layers by using the equation: E_VB_ = E_F_ - E_F_, edge (Fermi edge). The band gap (E_g_) values of TiO_2_ are obtained from the absorption spectrum and corresponding Tauc plot, showing negligible change of E_g_ from 3.20 and 3.22 eV for different TiO_2_ layers. Then the calculated conduction bands (E_CB_) calculated by E_g_ and E_VB_ are -4.22 and -4.12 eV for different TiO_2_ layers, respectively.


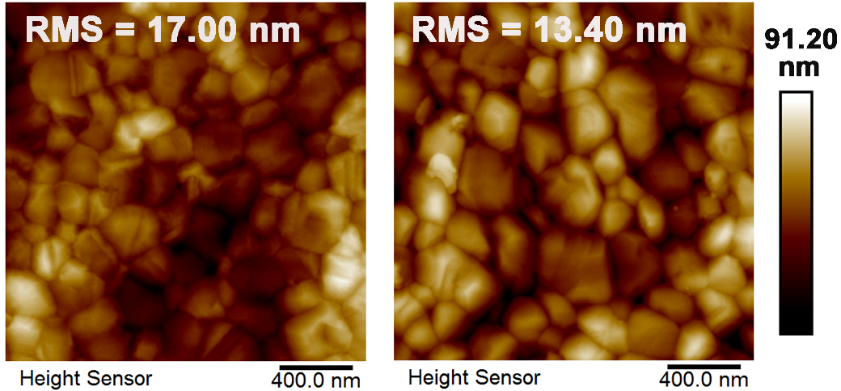


**Fig. S13** AFM images of CsFAMA perovskite based on **a** the pristine TiO_2_ and **b** 6%-target TiO_2_ layers


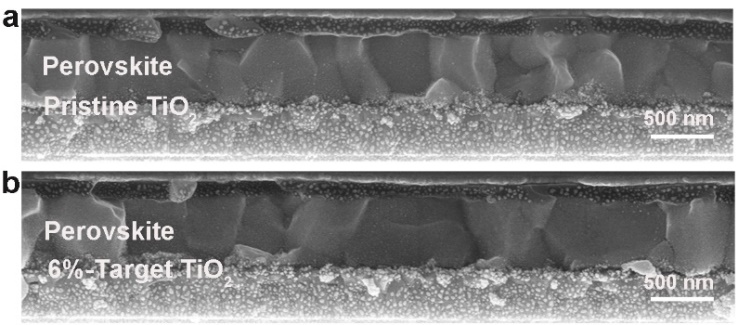


**Fig. S14** Cross-sectional SEM images of different devices


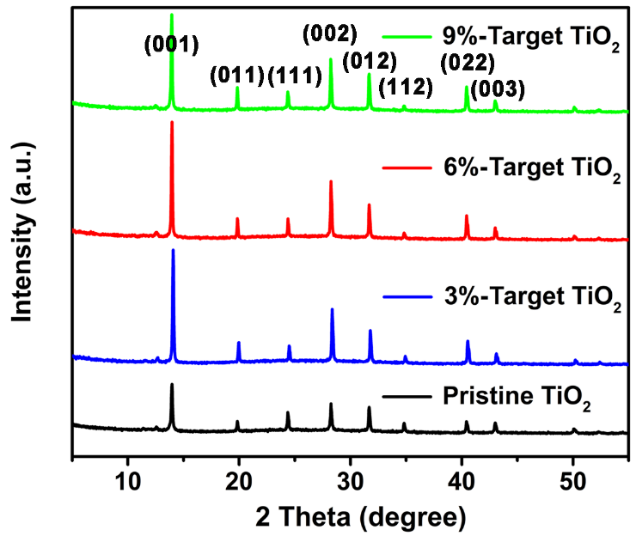


**Fig. S15** XRD patterns of CsFAMA perovskite upon different TiO_2_ layers. the improved crystallinity results from gradual increment of intensity for (001) diffraction plane of XRD patterns

**Table S2** Summaries of parameters extracted from the fitted plots of the corresponding TRPL spectra following bi-exponential rate law: f(t) = *A*_1_exp(-t/*τ*_1_ )+*A*_2_exp(-t/*τ*_2_ )+y_0_, where *A*_1_ and *A*_2_ represent the relative amplitudes, *τ*_1_ represents trap-assisted recombination, and *τ*_2_ represents free carrier recombination. The *τ*_ave_ is calculated using *τ*_ave_ = $\frac{\sum A_{i}\tau i^{2}}{\sum A_{i}\tau i}$ formula [S4]

| ETLs | *A*_1_ | *τ*_1_ (ns) | *A*_2_ | *τ*_2_ (ns) | *τ*_ave_ (ns) |
| --- | --- | --- | --- | --- | --- |
| Pristine TiO_2_ | 0.40 | 7.64 | 0.60 | 135.26 | 130.63 |
| 6%-Target TiO_2_ | 0.35 | 6.00 | 0.65 | 39.92 | 37.38 |

**Note S1: AS and Mott-Schottky Analyses**

Considering the limitations of the space charge-limited current (SCLC) on defect analysis, temperature dependent admittance spectroscopy (AS) along with Mott-Schottky analysis are adopted to quantitatively analyse both shallow and deep defects in PSCs. According to the previous literature [5], the defect activation energies (*E*_a_) is obtained by the equation:

$$\omega_{0}=\beta T^{2}exp(\frac{-E_{a}}{K_{b}T})$$

where $\omega_{0}$is the characteristic transition angular frequency, *β* is a temperature dependent parameter, T is the temperature and$K_{b}$is the Boltzmann’s constant, respectively. The derivative of the capacitance spectrum can be calculated to obtain $\omega_{0}$ from the peak value of the [$-\omega\times dC/d\omega$] curve. The value of$E_{a}$can be thus obtained from the slope of the plot using the Arrhenius formula [S5]:

$$\ln\frac{\omega_{0}}{T^{2}}=\ln\beta-\frac{E_{a}}{K_{b}T}$$

the trap density (*N*_t_) can be obtained according to the equation [S5]:

$N_{t}(E_{\omega})=-\frac{V_{bi}}{qW}\frac{dC}{d\omega}\frac{\omega}{k_{b}T}$ , $E_{\omega}=K_{b}T\ln\frac{\beta T^{2}}{\omega}$

where *V*_bi_ is the built-in potential, *C* is the capacitance, *ω* is the applied angular frequency, *q* is the elementary charge, and *W* is the depletion width, respectively. The values of *V*_bi_ and *W* were extracted from the Mott-Schottky [S5]:

$$\frac{A^{2}}{C^{2}}=\frac{2(V_{bi}-V)}{q\varepsilon\varepsilon_{0}N}$$

where *A* is the active area, $\varepsilon$ is the static permittivity of perovskite, $\varepsilon_{0}$ is the permittivity of free space, *N* is the apparent doping profile in the depleted layer, and *V* is the applied bias. The Mott-Schottky plot shows the intersection of a line with the bias axis for obtaining *V*_bi_ and the impurity doping density N is extracted from the slope of the line. Then, the depletion width $W=\sqrt{\frac{2\varepsilon\varepsilon_{0}V_{bi}}{qN}}$ that corresponds to the zero bias can be calculated, the distribution of trap state density can be further obtained. **Fig. S16a**, **b** show the capacitance-frequency (*C-f*) curves of the devices upon different ETLs, which were measured at temperatures ranging from 210 to 320 K under the dark. The defect activation energies (*E*_a_) of different devices are extracted from the corresponding Arrhenius plots that depicts the relationship between the characteristic transition frequencies and temperatures (**Fig. 2e**), which are calculated to be 0.277 and 0.223 eV, respectively. The built-in potential (*V*_bi_) and the depletion width (*W*) are derived from the Mott-Schottky, which were measured at a frequency of 1 kHz with bias potential from 0 to 1.2 V (**Fig. S16c**). The *V*_bi_ are determined to be 0.90 and 0.99 V for the control and the target, respectively, and the depletion widths are calculated to be 183 and 285 nm, respectively. The density distribution and energy levels of trap states of different devices are then showed in **Fig. 2f**.


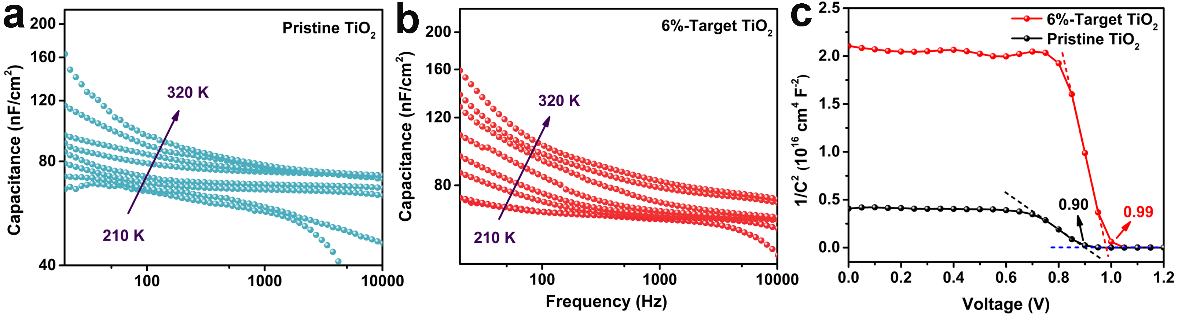


**Fig. S16** Admittance spectra of devices based on **a** pristine TiO_2_ and **b** 6%-target TiO_2_ ETLs were measured at gradient temperatures ranging from 210 to 320 K with a step of 15 K under the dark. **c** Mott-Schottky analysis at 1 kHz for obtaining the *V*_bi_





**Fig. S17** Dark current density-voltage plots of devices based on different TiO_2_ layers

**Table S3** Summaries of parameters extracted from the fitted plots of the impedance spectra of devices upon differnent ETLs using a the equivalent circuit model

| ETLs | *R*s  (Ω) | *R*co  (Ω) | CPE1-T  (F) | CPE1-P  (F) | *R*rec  (Ω) | CPE2-T  (F) | CPE2-P  (F) |
| --- | --- | --- | --- | --- | --- | --- | --- |
| Pristine TiO_2_ | 15.55 | 16544 | 5.16E-09 | 0.98 | 1.05E05 | 1.45E-06 | 0.81 |
| 6%-Target TiO_2_ | 7.65 | 12300 | 6.28E-09 | 0.97 | 1.64E05 | 1.27E-06 | 0.80 |


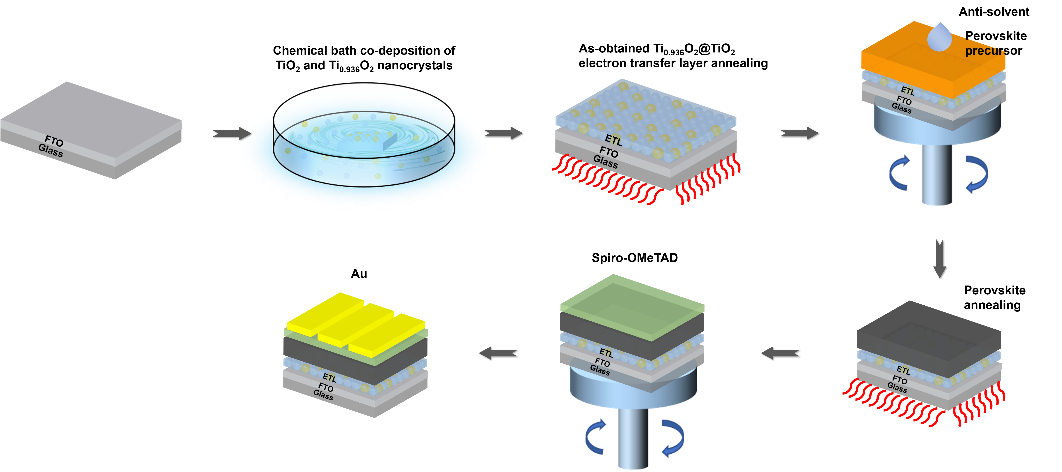


**Fig. S1****8** Schematic illustration of fabrication process of perovskite devices with Ti_0.936_O_2_@TiO_2_ ETLs

**Table S4** The Photovoltaic parameters of champion devices with and without defected TiO_2_ nanocrystals embedding under forward and reverse scanning

| Scanning  direction | *V*_OC_  (V) | *J*_SC_  (mA/cm^2^) | FF  (%) | PCE  (%) |
| --- | --- | --- | --- | --- |
| Forward control | 1.133 | 22.97 | 66.55 | 17.32 |
| Reverse control | 1.149 | 23.16 | 74.93 | 19.94 |
| Forward Target | 1.167 | 23.77 | 78.24 | 21.70 |
| Reverse Target | 1.194 | 23.72 | 77.75 | 22.02 |


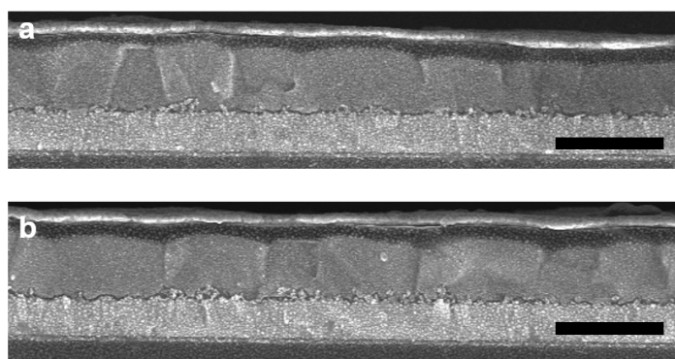


**Fig. S19** Cross-sectional SEM images of control FAPbI_3_ **a** and target FAPbI_3_ **b** devices, respectively. Scale Bar: 1μm


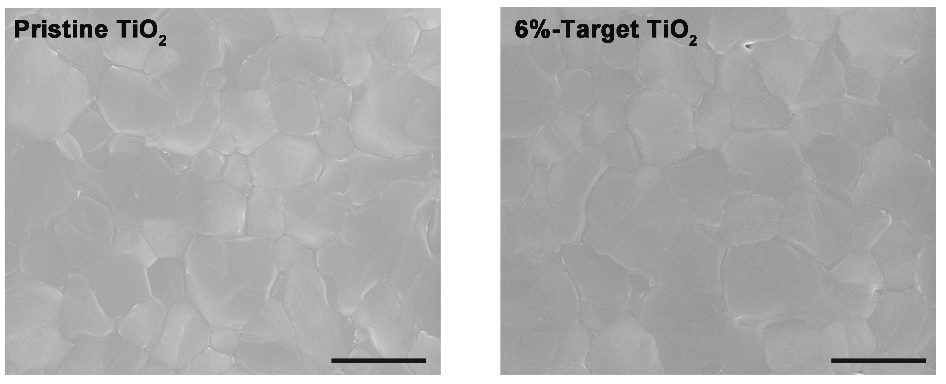


**Fig. S20** SEM images of FAPbI_3_ perovskite based on the pristine TiO_2_ **a** and target TiO_2_ **b** layers. Scale bars: 2 µm


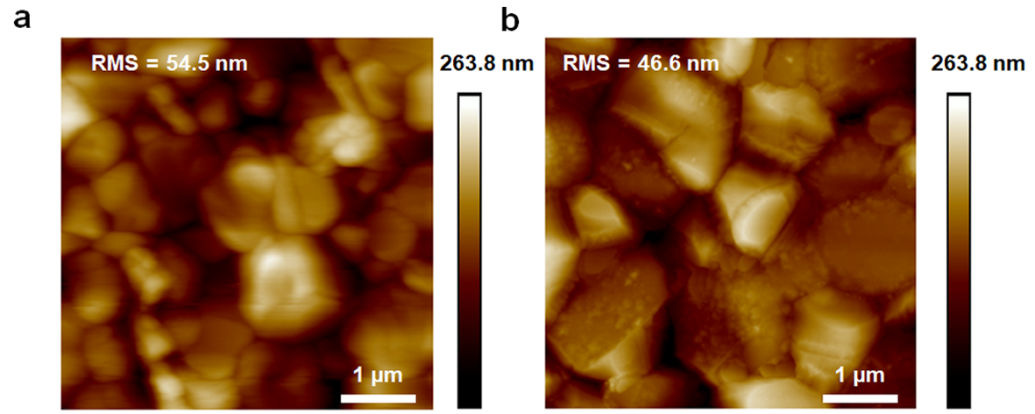


**Fig. S21** AFM images of FAPbI_3_ perovskite based on **a** the pristine TiO_2_ and **b** 6%-target TiO_2_ layers. Scale bars: 1 µm





**Fig. S22** XRD patterns of FAPbI_3_ perovskite upon different TiO_2_ layers


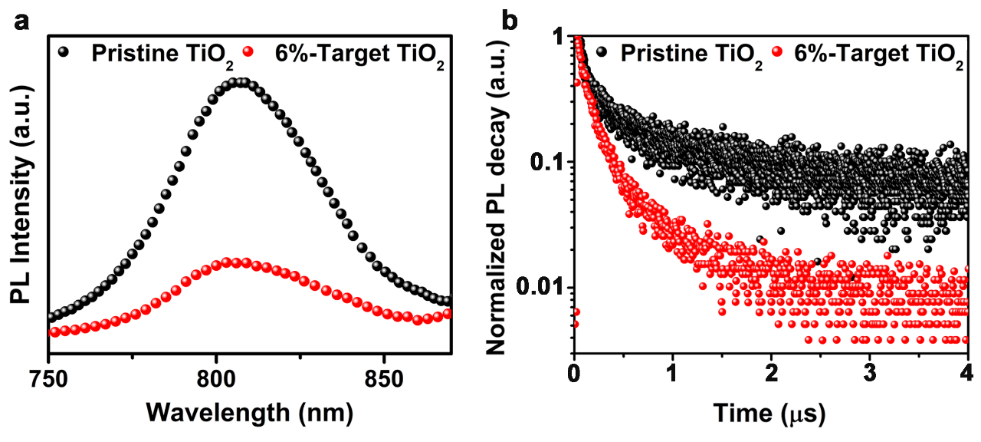


**Fig. S23** PL **a** and TRPL **b** spectra of FAPbI_3_ perovskite based on the pristine TiO_2_ and target TiO_2_ layers

**Table S5** Summaries of fitting parameters for TRPL spectra with a structure of FAPbI_3_/TiO_2_ (with and without defected TiO_2_ nanocrystals)/FTO substrates

| Samples | *A*_1_ | *Ʈ*_1_ (ns) | *A*_2_ | *Ʈ*_2_ (ns) | *Ʈ*_ave_ (ns) |
| --- | --- | --- | --- | --- | --- |
| FAPbI_3_ | 0.25 | 204.80 | 0.75 | 1311.17 | 1256.42 |
| Target FAPbI_3_ | 0.21 | 154.90 | 0.79 | 845.45 | 813.38 |





**Fig. S24** The Tauc plot of FAPbI_3_ film for obtaining bandgap


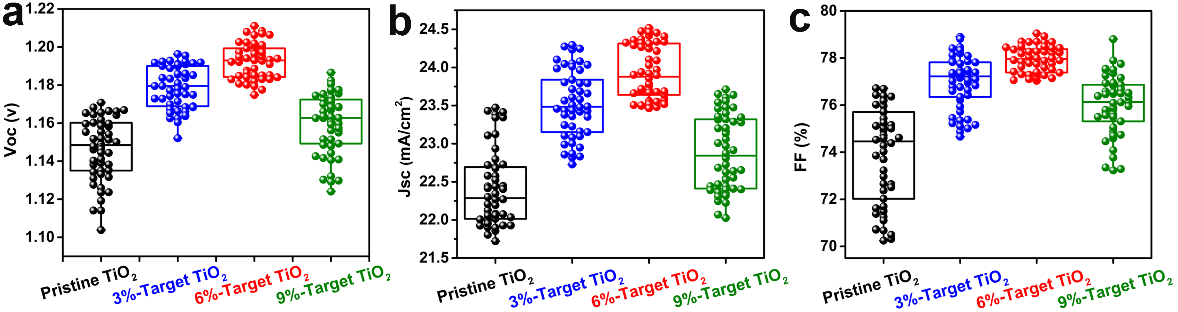


**Fig. S25** Statistical distributions of the photovoltaic parameters of *V*oc, *J*sc and FF for CsFAMA devices with different TiO_2_ layers. The average PCE improves from 18.28% to 21.22%, as shown in Figure 3e, with *V*oc from 1.147 to 1.192 V, the average *J*sc from 22.41 to 23.93 mA/cm^2^, and the FF from 73.88 to 77.93%


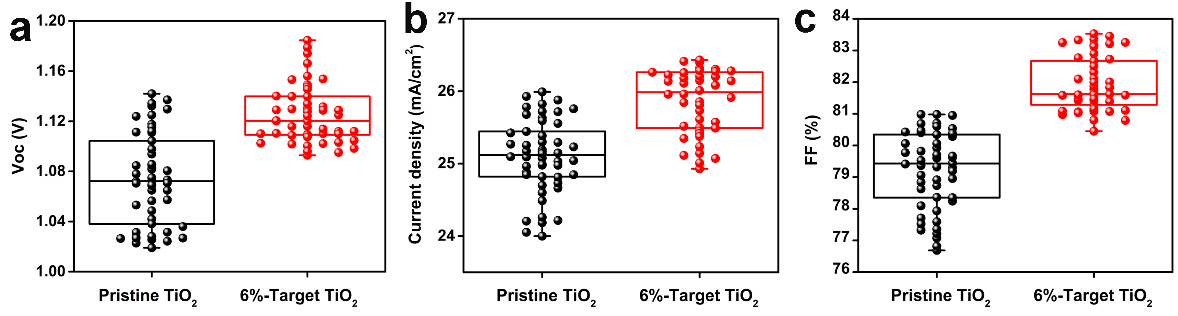


**Fig. S26** Statistical distributions of the photovoltaic parameters of *V*oc, *J*sc and FF for FAPbI_3_ devices based on different TiO_2_ layers. The average PCE increases from 21.24% to 23.52%, with *V*oc from 1.074 to 1.125 V, the average *J*sc from 25.10 to 25.86 mA/cm^2^, and the FF from 79.23 to 81.92%

**Table S6** Reported values for the efficiency of different PSCs based on TiO_2_ ETLs

| Type | Device  structure | Best PCE  (%) | Refs. |
| --- | --- | --- | --- |
| [BMIM]BF_4_ | FTO/TiO_2_/[BMIM]BF_4_/MAPbI_3_/PTAA/Au | 19.62 | [S6] |
| GQD | FTO/TiO_2_@GQD/MAPbI_3_/Spiro- OMeTAD/Au | 19.11 | [S7] |
| CuI | FTO/TiO_2_@CuI/CsFAMA/Spiro-OMeTAD/Au | 19.00 | [S8] |
| Heparin sodium | FTO/TiO_2_/heparin sodium/MAPbI_3_/Spiro-OMeTAD/Au | 20.1 | [S9] |
| Dopamine | FTO/Dopamine-TiO_2_/Cs_0.05_FA_0.81_MA_0.14_PbI_2.55_Br_0.45_/Spiro-OMeTAD/Au | 20.93 | [S10] |
| Urea | ITO/TiO_2_-urea/Cs_0.05_(FA_0.87_MA_0.13_)_0.95_PbI_2.55_Br_0.45_/Spiro-OMeTAD/Au | 21.33 | [S11] |
| Boron | FTO/Boron-TiO_2_/ MAPbI_3_/Spiro- OMeTAD/Au | 20.51 | [S12] |
| Li_2_CO_3_ | FTO/Li_2_CO_3_-TiO_2_/FAPbI_3_/Spiro- OMeTAD/Au | 25.28 | [S13] |
| TiO_x_N_y_ | FTO/TiO_x_N_y_/TiO_2_/PMMA:PCBM/ Cs_0.05_FA_0.88_MA_0.07_PbI_2.56_Br_0.44_/PMMA/P3HT:CuPC/Au | 23.28 (1cm^2^) | [S14] |
| Single-crystalline TiO_2_ | FTO/Single-crystalline TiO_2_/ Rb_0.03_Cs_0.05_MA_0.05_FA_0.9_PbI_3_/Spiro- OMeTAD/Au | 24.05 | [S15] |
| Tartaric acid | FTO/tartaric acid-TiO_2_/FAMAPbI_3_/Spiro-OMeTAD/Au | 24.81 | [S16] |
| C-PCBA | FTO/TiO_2_/C-PCBA/CsFAPb(IBr)_3_/Spiro-OMeTAD/Au | 24.80 | [S17] |
| CdTe nanocrystals | [FTO/TiO_2_@CdTe/Cs_0.05_(FA_0.85_MA_0.15_)_0.95_PbI_2.55_Br_0.45_](mailto:FTO/TiO2@CdTe/Cs0.05(FA0.85MA0.15)0.95PbI2.55Br0.45)/Spiro-OMeTAD/Au | 22.00 | [S4] |
| CdTe nanocrystals | FTO/TiO_2_@CdTe/FAPbI_3_/Spiro-OMeTAD/Au | 25.05 | [S4] |
| Ti_0.936_O_2_ | [FTO/TiO_2_@ Ti_0.936_O_2_/Cs_0.05_(FA_0.85_MA_0.15_)_0.95_PbI_2.55_Br_0.45_](mailto:FTO/TiO2@CdTe/Cs0.05(FA0.85MA0.15)0.95PbI2.55Br0.45)/Spiro-OMeTAD/Au | 22.02 | Our present work |
| Ti_0.936_O_2_ | FTO/TiO_2_@ Ti_0.936_O_2_/FAPbI_3_/Spiro-OMeTAD/Au | 25.50 | Our present work |


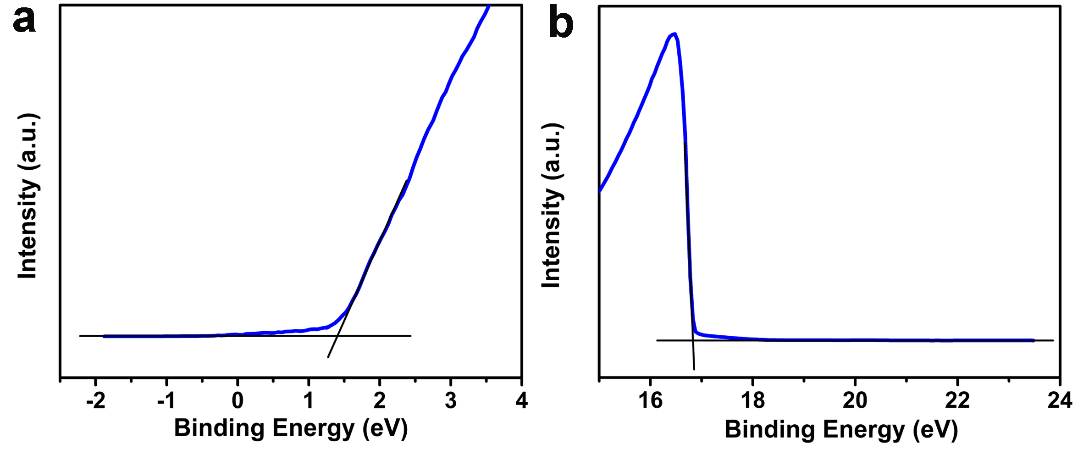


**Fig. S27** UPS Fermi edge **a** and the cut-off energy **b** of CsFAMA perovskite film. The calculation of the E_F_, E_VB_ and E_CB_ of CsFAMA-based perovskite film are -4.35, -5.70 and -4.10 eV


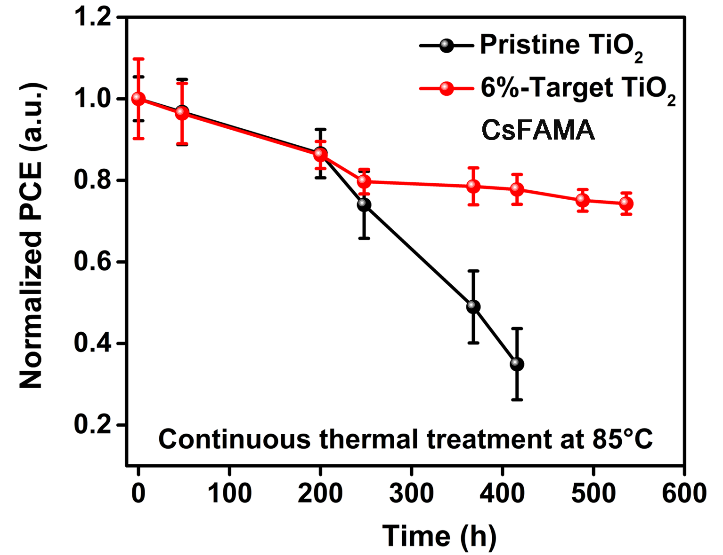


**Fig. S28** The long-term thermal stability of CsFAMA-based devices with and without Ti_0.936_O_2_ nanocrystals embedding. The excellent thermal stability of target devices presents PCE degradation of less than 30% over 500 h in contrast with that of control devices (over 60%) after continuous thermal treatment of 85 °C. The error bars represent the standard deviation for 20 devices.


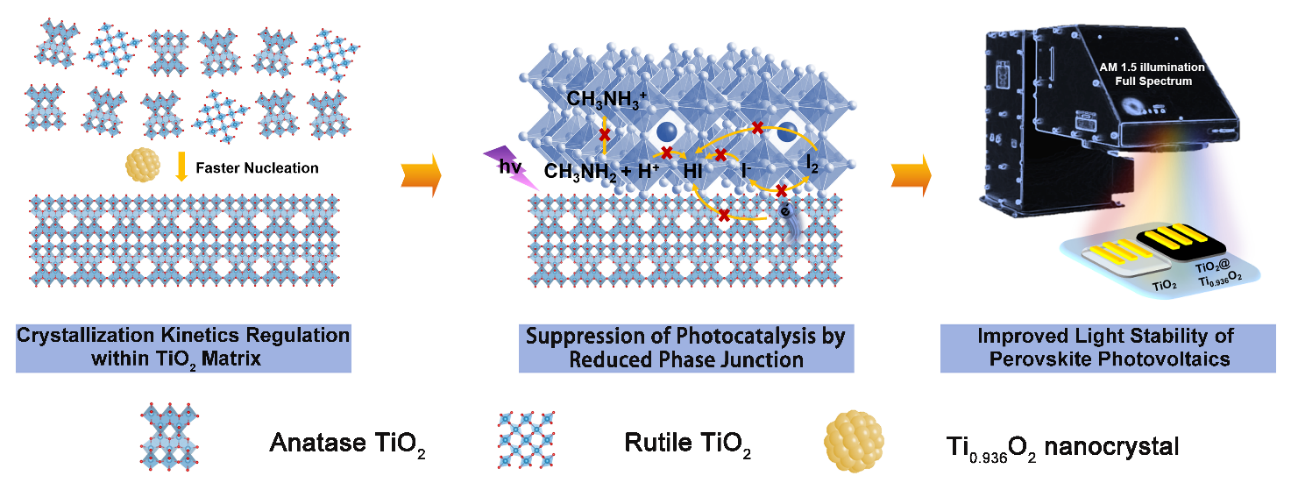


**Fig. S29** Schematic illustration of the effects of embedding Ti_0.936_O_2_ nanocrystals on crystallization kinetics and light-induced stability





**Fig. S30** *J-V* curves of the champion FAPbI_3_ devices employing 6%-Target TiO_2_ layers measured both in reverse scan and forward scan

**Table S7** Performance parameters of champion FAPbI_3_ devices employing 6%-Target TiO_2_ layers measured both in reverse scan and forward scan

| Scanning direction | *V*oc  (V) | *J*sc  (mA/cm^2^) | FF  (%) | PCE  (%) |
| --- | --- | --- | --- | --- |
| Reverse  6%-Target TiO_2_ | 1.185 | 25.79 | 83.45 | 25.50 |
| Forward  6%-Target TiO_2_ | 1.183 | 25.68 | 82.43 | 25.04 |

The hysteresis index is calculated to be 0.018 according the equation below:

Hysteresis index = (PCE_Reverse_-PCE_Forward_)/ PCE_Reverse_

**Supplementary References**

1. H. Yu, W. Zhao, L. Ren, H. Wang, P. Guo et al., Laser-generated supranano liquid metal as efficient electron mediator in hybrid perovskite solar cells. Adv. Mater. **32**, e2001571 (2020). <https://doi.org/10.1002/adma.202001571>
2. J. Zhang, M. Li, Z. Feng, J. Chen, C. Li UV Raman spectroscopic study on TiO_2_. I. phase transformation at the surface and in the bulk. J. Phys. Chem. B **110**, 927–935 (2006). <https://doi.org/10.1021/jp0552473>
3. S. Wang, L. Pan, J.-J. Song, W. Mi, J.-J. Zou et al., Titanium-defected undoped anatase TiO_2_ with p-type conductivity, room-temperature ferromagnetism, and remarkable photocatalytic performance. J. Am. Chem. Soc. **137**, 2975–2983 (2015). <https://doi.org/10.1021/ja512047k>
4. W. Zhao, P. Guo, C. Liu, N. Jia, Z. Fang et al., Laser derived electron transport layers with embedded p-n heterointerfaces enabling planar perovskite solar cells with efficiency over 25. Adv. Mater. **35**, e2300403 (2023). <https://doi.org/10.1002/adma.202300403>
5. N. Li, S. Tao, Y. Chen, X. Niu, C.K. Onwudinanti et al., Cation and anion immobilization through chemical bonding enhancement with fluorides for stable halide perovskite solar cells. Nat. Energy **4**, 408–415 (2019). <https://doi.org/10.1038/s41560-019-0382-6>
6. D. Yang, X. Zhou, R. Yang, Z. Yang, W. Yu et al., Surface optimization to eliminate hysteresis for record efficiency planar perovskite solar cells. Energy Environ. Sci. **9**, 3071–3078 (2016). <https://doi.org/10.1039/C6EE02139E>
7. J. Ryu, J.W. Lee, H. Yu, J. Yun, K. Lee et al., Size effects of a graphene quantum dot modified-blocking TiO_2_ layer for efficient planar perovskite solar cells. J. Mater. Chem. A **5**, 16834–16842 (2017). <https://doi.org/10.1039/C7TA02242E>
8. M.M. Byranvand, T. Kim, S. Song, G. Kang, S.U. Ryu et al., P-type CuI islands on TiO_2_ electron transport layer for a highly efficient planar-perovskite solar cell with negligible hysteresis. Adv. Energy Mater. **8**, 1702235 (2018). <https://doi.org/10.1002/aenm.201702235>
9. S. You, H. Wang, S. Bi, J. Zhou, L. Qin et al., A biopolymer heparin sodium interlayer anchoring TiO_2_ and MAPbI_3_ enhances trap passivation and device stability in perovskite solar cells. Adv. Mater. **30**, e1706924 (2018). <https://doi.org/10.1002/adma.201706924>
10. Y. Zhang, X. Liu, P. Li, Y. Duan, X. Hu et al., Dopamine-crosslinked TiO_2_/perovskite layer for efficient and photostable perovskite solar cells under full spectral continuous illumination. Nano Energy **56**, 733–740 (2019). <https://doi.org/10.1016/j.nanoen.2018.11.068>
11. W. Hu, W. Zhou, X. Lei, P. Zhou, M. Zhang et al., Low-temperature *in situ* amino functionalization of TiO_2_ nanoparticles sharpens electron management achieving over 21% efficient planar perovskite solar cells. Adv. Mater. **31**, 1806095 (2019). <https://doi.org/10.1002/adma.201806095>
12. X. Shi, Y. Ding, S. Zhou, B. Zhang, M. Cai et al., Enhanced interfacial binding and electron extraction using boron-doped TiO_2_ for highly efficient hysteresis-free perovskite solar cells. Adv. Sci. **6**, 1901213 (2019). <https://doi.org/10.1002/advs.201901213>
13. M. Kim, I.-W. Choi, S.J. Choi, J.W. Song, S.-I. Mo et al., Enhanced electrical properties of Li-salts doped mesoporous TiO_2_ in perovskite solar cells. Joule **5**, 659–672 (2021). <https://doi.org/10.1016/j.joule.2021.02.007>
14. J. Peng, F. Kremer, D. Walter, Y. Wu, Y. Ji et al., Centimetre-scale perovskite solar cells with fill factors of more than 86 per cent. Nature **601**, 573–578 (2022). <https://doi.org/10.1038/s41586-021-04216-5>
15. Y. Ding, B. Ding, H. Kanda, O.J. Usiobo, T. Gallet et al., Single-crystalline TiO_2_ nanoparticles for stable and efficient perovskite modules. Nat. Nanotechnol. **17**, 598–605 (2022). <https://doi.org/10.1038/s41565-022-01108-1>
16. H. Huang, P. Cui, Y. Chen, L. Yan, X. Yue et al., 24.8%-efficient planar perovskite solar cells via ligand-engineered TiO_2_ deposition. Joule **6**, 2186–2202 (2022). <https://doi.org/10.1016/j.joule.2022.07.004>
17. Z. Chen, Y. Li, Z. Liu, J. Shi, B. Yu et al., Reconfiguration toward self-assembled monolayer passivation for high-performance perovskite solar cells. Adv. Energy Mater. **13**, 2202799 (2023). <https://doi.org/10.1002/aenm.202202799>
